# Exploration of the Dynamic Variations of the Characteristic Constituents and the Degradation Products of Catalpol during the Process of Radix Rehmanniae

**DOI:** 10.3390/molecules29030705

**Published:** 2024-02-03

**Authors:** Jingjing Yang, Lihua Zhang, Mengyue Zhang, Mingxuan Yang, Lin Zou, Ying Cui, Jing Yang, Xin Chai, Yuefei Wang

**Affiliations:** 1 National Key Laboratory of Chinese Medicine Modernization, State Key Laboratory of Component-Based Chinese Medicine, Tianjin Key Laboratory of TCM Chemistry and Analysis, Tianjin University of Traditional Chinese Medicine, Tianjin 301617, China; yangpgjj@163.com (J.Y.); 16622765055@163.com (L.Z.); zhangmengyue1113@163.com (M.Z.); 15093240403@163.com (M.Y.); linzou230505@126.com (L.Z.); cql8179270@tjutcm.edu.cn (Y.C.); yangjingoffice@163.com (J.Y.); 2 Haihe Laboratory of Modern Chinese Medicine, Tianjin 301617, China

**Keywords:** Radix Rehmanniae, catalpol, degradation products, processing, UPLC-PDA

## Abstract

Radix Rehmanniae (RR), a famous traditional Chinese medicine (TCM) widely employed in nourishing *Yin* and invigorating the kidney, has three common processing forms in clinical practice, including fresh Radix Rehmanniae (FRR), raw Radix Rehmanniae (RRR), and processed Radix Rehmanniae (PRR). However, until now, there has been less exploration of the dynamic variations in the characteristic constituents and degradation products of catalpol as a representative iridoid glycoside with the highest content in RR during the process from FRR to PRR. In this study, an ultra-performance liquid chromatography coupled with photodiode array detector (UPLC-PDA) method was successfully established for the simultaneous determination of ten characteristic components to explore their dynamic variations in different processed products of RR. Among them, iridoid glycosides, especially catalpol, exhibited a sharp decrease from RRR to PRR. Then, three degradation products of catalpol were detected under simulated processing conditions (100 °C, pH 4.8 acetate buffer solution), which were isolated and identified as jiofuraldehyde, cataldehyde, and norviburtinal, respectively. Cataldehyde was first reported as a new compound. Moreover, the specificity of norviburtinal in self-made PRR samples was discovered and validated, which was further confirmed by testing in commercially available PRR samples. In conclusion, our study revealed the decrease in iridoid glycosides and the production of new degradation substances during the process from FRR to PRR, which is critical for unveiling the processing mechanism of RR.

## 1. Introduction

*Rehmannia glutinosa* Libosch., a member of the Scrophulariaceae family, is widely distributed in different regions of China, such as Henan, Hebei, and Shanxi Provinces [1]. Radix Rehmanniae (RR) is the root of *R. glutinosa* [2], which is classified into the top grade by *Shennong’s Classic of Materia Medica*. RR is employed in a variety of famous traditional Chinese medicine (TCM) prescriptions, such as Liuwei Dihuang Pill, Zhibai Dihuang Pill, Qiju Dihuang Pill, and Dihuang Yinzi, owing to its ability to nourish *Yin* and tonify the kidney [3,4]. There are three processing forms frequently used in clinic, including fresh Radix Rehmanniae (FRR), raw Radix Rehmanniae (RRR), and processed Radix Rehmanniae (PRR), with their pharmacological actions varying greatly from heat-clearing to tonification [5].

There must be chemical variations behind the change in their efficacy, giving rise to different clinical applications. At present, hundreds of chemical constituents have been isolated and identified from *R. glutinosa*, including iridoids, ionones, phenylethanoid glycosides, triterpenoids, flavonoids, saccharides, amino acids, and so on [6,7]. It was also reported that the degradation of certain components, such as catalpol (Cat), leonuride (Leo), rehmannioside A, rehmannioside D (RhD), stachyose, raffinose, sucrose, and amino acids, and the formation of fructose, glucose, and 5-hydroxymethylfurfural synchronously occurred in the process from RRR to PRR [8,9]. As the characteristic compounds of iridoids, Cat is stipulated as the indicator for the quality evaluation of RRR by the Chinese pharmacopoeia (ChP), which has been proven to exhibit cardioprotective [10], anti-osteoporotic [11], anti-atherosclerotic [12], anti-inflammatory [13], antidiabetic [14], neuroprotective [15], and liver protective effects [16]. However, it is hard to detect Cat in PRR, suggesting the occurrence of a series of hydrolysis and polymerization reactions during the steaming process [17]. Researchers investigated the degradation characteristics of Cat under different exposure conditions [18,19]. However, the kinds of degradation products generated from Cat have not yet been studied.

Processing plays a crucial role in the quality and application of PRR. However, specific processing parameters such as processing cycles, steaming time, and subsidiary material are not yet clear. Moreover, it is too subjective to determine the endpoint of the preparation of PRR by the criterion of “color black as lacquer, sweet as candy” [20]. At the same time, it is infeasible to distinguish RRR and PRR only through appearance identification by virtue of their similar colors, giving rise to misuse and failure to achieve their clinical efficacy [21]. Therefore, it is necessary to search for a specific chemical marker to distinguish RRR from PRR.

In this study, we focused on dynamic variations of representative compounds in the process from FRR to PRR, including iridoid glycosides, nucleosides, and phenylethanol glycosides, using ultra-performance liquid chromatography with the photo-diode array detector (UPLC-PDA) method, which was first investigated. Among them, iridoid glycosides gained our attention because their contents presented a sharp decrease from RRR to PRR, especially Cat. Then, three degradation products of Cat were successfully discovered and elucidated under simulated processing conditions (100 °C, pH 4.8 acetate buffer solution). Cataldehyde, one of the degradation products from Cat, was reported as a new compound. Moreover, the specificity of degradation products of Cat was explored by comparing RRR and PRR samples. This paper aims to demonstrate that quantitative and qualitative changes occur in the process from FRR to PRR, which will contribute to illumination on the processing-induced chemical transformations in RR, providing a reliable reference for the development of quality standards for PRR.

## 2. Results and Discussions

### 2.1. Methodological Validation of UPLC-PDA Analysis for Quantitation of Ten Compounds in RR

**Figure 1:**
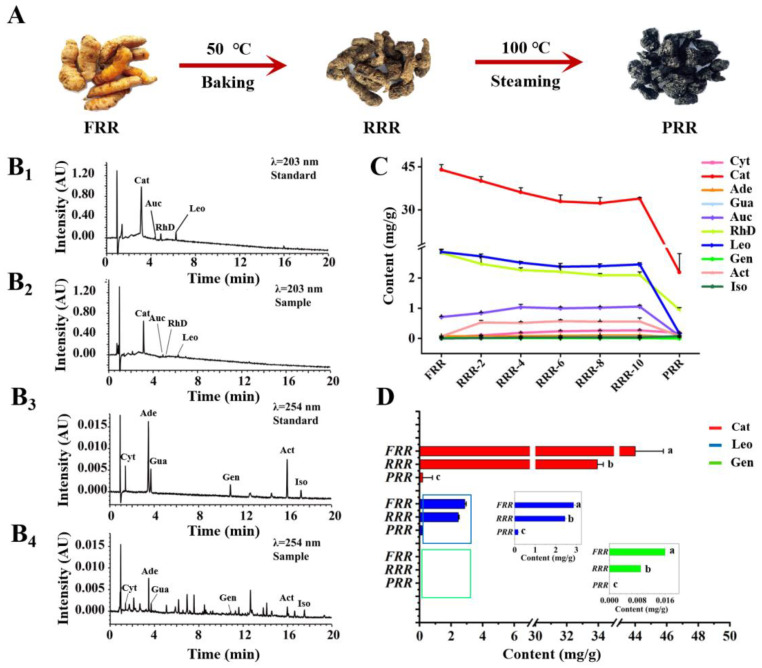
Dynamic changes in appearance and contents of the detected compounds during the process from FRR to PRR. (**A**) Variations in the appearance in the process from FRR to PRR. UPLC-PDA chromatograms of the mixed standard solution (**B_1_**) and sample solution (**B_2_**) under the detection wavelength of 203 nm (for Cat, Auc, RhD, and Leo), and the mixed standard solution (**B_3_**) and sample solution (**B_4_**) under the detection wavelength of 254 nm (for Cyt, Ade, Gua, Gen, Act, and Iso). (**C**) Dynamic variations in the detected compounds’ contents from FRR to PRR. (**D**) The histograms of the contents of Cat, Leo, and Gen in FRR, RRR, and PRR. Different letters indicated that data were significantly different at *p* < 0.05 when ANOVA analysis was applied. Abbreviation notes: FRR: fresh Radix Rehmanniae; RRR: raw Radix Rehmanniae; PRR: processed Radix Rehmanniae; Cyt: cytidine; Cat: catalpol; Ade: adenosine; Gua: guanosine; Auc: aucubin; RhD: rehmannioside D; Leo: leonuride; Gen: geniposide; Act: acteoside; Iso: isoacteoside.

As shown in Figure 1A, after baking at 50 °C and steaming at 100 °C, the color of RR samples gradually darkened and the texture became sticky. A rapid UPLC-PDA quantification was further developed to investigate content changes behind the variations in appearance. To improve the resolution and sensitivity of the tested compounds, gradient elution, column temperature, and detection wavelength were systematically optimized. In addition, the sample preparation method of ten compounds was optimized by investigating the extraction solvent, extraction temperature, material/solvent ratio, and ultrasonic time to achieve the highest extracting efficiency (see Supplementary Materials, Table S1). The typical chromatograms of the sample and mixed standard solution are displayed in Figure 1(B_1_–B_4_).

The systematical method validation was carried out in terms of linearity, the limit of detection (LOD), the limit of quantitation (LOQ), precision, repeatability, stability, and recovery. As shown in Table 1, the calibration curves of ten compounds were established with the determination coefficient (*r*^2^) exceeding 0.9990, indicating a good linear correlation within the tested ranges. The LOD and LOQ values were in the range of 0.0138–9.3334 µg/mL and 0.0414–28.000 µg/mL, respectively. The relative standard deviation (RSD) values of intra- and inter-day precisions were less than 2.6% and 3.0%, respectively. Repeatability and stability were evidenced to be acceptable with RSD values below 2.9% and 2.8%. The mean recovery was in the range of 91.5–101.6%, with RSD values below 4.9%. In summary, all these results showed the stability, accuracy, and feasibility of the established UPLC-PDA method.

### 2.2. The Content Variations of Tested Compounds and the Degradation of Cat That Occurred in the Processing of RR

The variations in the characteristic components in the procedure of processing are related to the efficacy differences in different processed products of TCMs. In this study, the contents of ten crucial components in different processed products of RR deriving from iridoid glycosides, nucleosides, and phenylethanol glycosides were determined using the established UPLC-PDA method. As displayed in Figure 1C, iridoid glycosides including Cat, RhD, aucubin (Auc), Leo, and geniposide (Gen) showed an increased or decreased tendency to different extents from FRR to RRR, while all of them presented a sharp decrease from RRR to PRR. Nevertheless, no obvious changes were witnessed in the contents of nucleosides and phenylethanol glycosides. From Figure 1D, Cat, Leo, and Gen represented by the epoxy ether[*c*]pyran, cyclopentano[*c*]pyran, and cyclopentenyl[*c*]pyran ring systems, respectively, were degraded gradually from FRR to RRR, yet rapidly destroyed from RRR to PRR. Among them, the most significant declining tendency was observed for Cat with its content decreasing from 44.00 mg/g in FRR to 2.192 mg/g in PRR.

**Figure 2:**
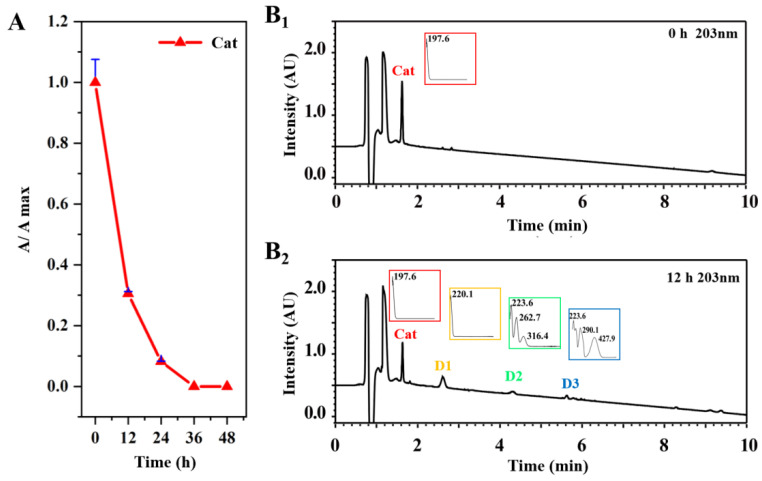
The discovery of the degradation products of Cat under simulated preparation conditions. (**A**) The dynamic variations in Cat content at different heating time points. Typical UPLC-PDA chromatograms of the degradation products of Cat for 0 h (**B_1_**) and 12 h (**B_2_**) under simulated processing conditions. Abbreviation notes: Cat: catalpol; **D1**: compound **D1**; **D2**: compound **D2**; **D3**: compound **D3**.

High temperature and long-term heating are indispensable for the processing of PRR, leading to the decomposition of the proteins into acidic peptides or free amino acids [22] and the formation of an acidic environment in the PRR. Therefore, Cat mainly exists as aglycone or its rearranged products instead of a prototype component in PRR [23]. The content variations of Cat at different heating time points are illustrated in Figure 2A. After 12 h, the content decreased remarkably to about 30% and even nearly disappeared after 36 h due to steaming. After heating for 12 h, the new peaks detected at three different retention times were recognized as unknown degradation products of Cat, which were referred to as **D1**–**D3** according to retention time, separately Figure 2(B1,B2). The discovery of degradation products is meaningful for clarification of the processing mechanism of RR.

### 2.3. Characterization of the Degradation Products of Cat

**Figure 3:**
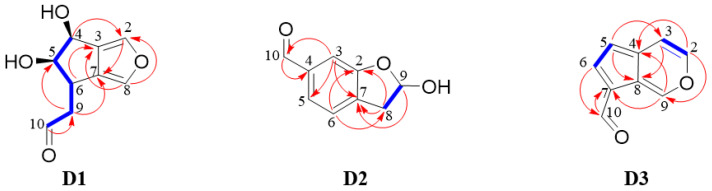
Key HMBC and ^1^H-^1^H COSY correlations of degradation products **D1**–**D3**.

In the current study, the isolation and purification of Cat degradation products were carried out using various methods, such as column chromatography (CC) with D101 macroporous resin and preparative high-performance liquid chromatography (preparative HPLC), and nuclear magnetic resonance (NMR) spectroscopic analysis was used for structure elucidation. Key heteronuclear multiple bond correlation (HMBC) and ^1^H-^1^H correlated spectroscopy (^1^H-^1^H COSY) correlations of compounds **D1**–**D3** are shown in Figure 3. ^1^H NMR (600 MHz) and ^13^C NMR (150 MHz) spectroscopic data of compounds **D1**–**D3** are presented in Table 2.

#### 2.3.1. Spectroscopic Analysis of **D1**

**D1** was obtained as a white amorphous powder. The HR-ESI-MS (negative-ion mode) exhibited a quasi-molecular ion peak at *m*/*z* 181.0487 ([M − H]^−^, calcd. for 181.0579) that corresponded to molecular formula C_9_H_10_O_4_, marking the presence of five degrees of unsaturation.

The ^1^H NMR spectrum of **D1** revealed an aldehyde proton signal at *δ*_H_ 9.82 (1H, t, *J* = 1.8 Hz, H-10) and a methylene group at *δ*_H_ 2.60 (1H, ddd, *J* = 1.8, 9.0, 17.4 Hz) and *δ*_H_ 2.85 (1H, ddd, *J* = 1.8, 5.4, 17.4 Hz). Three signals at *δ*_H_ 4.60 (1H, d, *J* = 4.8 Hz), *δ*_H_ 3.79 (1H, d, *J* = 4.8, 8.4 Hz), and *δ*_H_ 3.15 (1H, m) were assigned to the methine protons at C-4, C-5, and C-6, respectively. Signals at *δ*_H_ 7.52 (1H, s) and *δ*_H_ 7.17 (1H, s) were attributed to two olefinic protons. The ^13^C NMR spectrum demonstrated nine carbon signals, including one carbonyl at *δ*_C_ 202.9 and four olefinic carbons at *δ*_C_ 136.0, 134.3, 130.5, and 130.2. ^1^H-^1^H COSY analysis demonstrated the existence of ‘-CH(4)-CH(5)-CH(6)-CH_2_(9)-CH(10)-’. The two five-member rings were fused by the key HMBC correlations of H-4 (*δ*_H_ 4.60) with C-2 (*δ*_C_ 136.0) and C-7 (*δ*_C_ 130.2), H-6 (*δ*_H_ 3.15) with C-3 (*δ*_C_ 130.5) and C-8 (*δ*_C_ 134.3), H-2 (*δ*_H_ 7.52) with C-7 (*δ*_C_ 130.2), and H-8 (*δ*_H_ 7.17) with C-3 (*δ*_C_ 130.5). Furthermore, the position of attachment of the aldehyde moiety to C-9 was deduced by the HMBC correlation observed between H-10 (*δ*_H_ 9.82) and C-9 (*δ*_C_ 45.8) (see Supplementary Materials, Figures S1–S6).

The signal patterns of the NMR spectra were similar to those of jiofuran isolated from steamed root of *Rehmannia glutinosa* var. *hueichingensis* [24], except that **D1** had an aldehyde moiety instead of the C-10 hydroxy methyl moiety in jofuran. Given that the chemical shifts and coupling constants of H-4 and H-5 were consistent with those reported in the literature, the stereo-configurations of H-4 and H-5 should be identical to those in the literature. The ^1^H NMR and ^13^C NMR spectral data were in line with published data [25], so **D1** was identified as jiofuraldehyde.

#### 2.3.2. Spectroscopic Analysis of **D2**

**D2** was obtained as a brown powder. The molecular formula was deduced to be C_9_H_8_O_3_ from HR-ESI-MS (negative-ion mode) with a quasi-molecular ion peak at *m*/*z* [M–H]^−^ 163.0398 (calcd. for 163.0473), which suggested six degrees of unsaturation.

The ^1^H NMR spectrum of **D2** revealed an aldehyde proton signal at *δ*_H_ 9.85 (1H, s, H-10), an oxymethine proton at *δ*_H_ 4.85 (1H, m, H-9), and a methylene group at *δ*_H_ 2.98 (1H, dd, *J* = 5.4, 13.8 Hz, H-8a) and *δ*_H_ 2.90 (1H, dd, *J* = 5.4, 13.8 Hz, H-8b). Signals at *δ*_H_ 7.27 (1H, s, H-3), 7.33 (1H, brs, H-5), and 7.33 (1H, s, H-6) were attributed to aromatic protons. The ^13^C NMR spectrum of **D2** showed nine resonating signals, comprising one carbonyl at *δ*_C_ 194.0 and six aromatic carbons at *δ*_C_ 157.6, 137.9, 133.4, 133.2, 123.1, and 115.1. In the ^1^H-^1^H COSY spectrum, H-8 (*δ*_H_ 2.98 and 2.90) protons showed coupling with H-9 (*δ*_H_ 4.85). In the HMBC spectrum, correlations were found between H-3 (*δ*_H_ 7.27) and C-7 (*δ*_C_ 133.2), H-6 (*δ*_H_ 7.33) and C-2 (*δ*_C_ 157.5), H-6 (*δ*_H_ 7.33) and C-8 (*δ*_C_ 39.6), and H-8 (*δ*_H_ 2.98 and 2.90) and C-2 (*δ*_C_ 157.5), indicating that the five- and six-member rings were fused by C-2 and C-7. Moreover, H-10 (*δ*_H_ 9.85) was associated with C-4 (*δ*_C_ 137.9), showing that the aldehyde group was connected to C-4 (See Supplementary Materials, Figures S7–S12). Thus, the structure of **D2** was deduced as 9-hydroxy-8,9-dihydrobenzofuran-4-carbaldehyde, designated as cataldehyde.

#### 2.3.3. Spectroscopic Analysis of **D3**

**D3** was isolated as a yellow powder. Its molecular formula is C_9_H_6_O_2_, from its HR-ESI-MS (positive-ion mode) with a quasi-molecular ion peak at *m*/*z* 147.0442 [M+H]^+^ (calcd. for 147.0368), indicating seven degrees of unsaturation.

The ^1^H NMR spectrum of **D3** showed an aldehyde proton signal at *δ*_H_ 9.66 (1H, s, H-10) and five olefinic protons at *δ*_H_ 8.11 (1H, d, *J* = 4.8 Hz, H-2), *δ*_H_ 7.56 (1H, d, *J* = 4.8 Hz, H-3), *δ*_H_ 6.71 (1H, d, *J* = 3.6 Hz, H-5), *δ*_H_ 8.09 (1H, d, *J* = 3.6 Hz, H-6), and *δ*_H_ 9.27 (1H, s, H-9). The relatively large chemical shifts of H-2 and H-9 and the small coupling constant of H-2 and H-3 supported the presence of an oxygen atom linking H-2 with H-9. Nine carbon signals were observed in the ^13^C NMR spectrum, including one carbonyl at *δ*_C_ 187.3 and eight olefin carbons. In the ^1^H-^1^H COSY spectrum, correlations were found between H-2 (*δ*_H_ 8.11) and H-3 (*δ*_H_ 7.56), and H-5 (*δ*_H_ 6.71) and H-6 (*δ*_H_ 8.09). The key HMBC correlations of H-9 (*δ*_H_ 9.27) with C-4 (*δ*_C_ 139.0) and C-7 (*δ*_C_ 123.2), H-2 (*δ*_H_ 8.11) with C-4 (*δ*_C_ 139.0), H-5 (*δ*_H_ 6.71) with C-3 (*δ*_C_ 112.3) and C-8 (*δ*_C_ 124.0), and H-3 (*δ*_H_ 7.56) with C-8 (*δ*_C_ 124.0) were observed, suggesting the two rings fused by C-4 and C-8. In addition, H-10 (*δ*_H_ 9.66) exhibited an interaction with C-7 (*δ*_C_ 123.2), which indicated that the aldehyde group was attached to C-7 (See Supplementary Materials, Figures S13–S18). Thus, the structure of **D3** was in line with published data, deduced as cyclopenta[*c*]pyran-7-carbaldehyde, named as norviburtinal [26,27].

### 2.4. Specificity of **D3** as a Degradation Product of Cat in PRR

**Figure 4:**
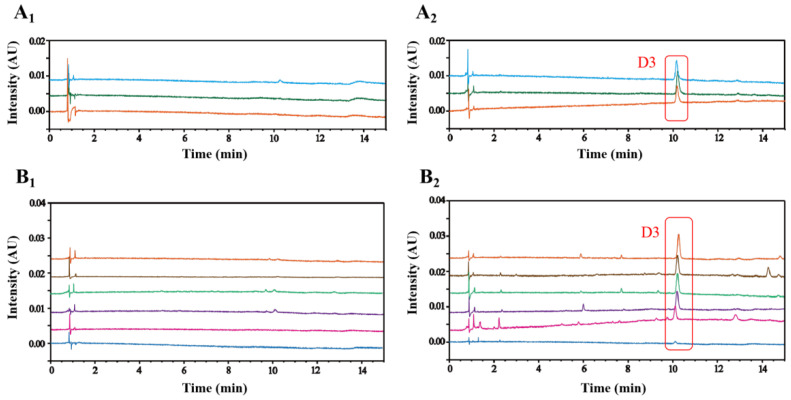
Validation of the specificity of **D3** in PRR. Validation of **D3** in three batches of RRR samples (**A_1_**) and their corresponding self-made PRR samples (**A_2_**). Validation of **D3** in commercially available RRR samples (**B_1_**) and PRR samples (**B_2_**).

In order to validate the specificity of Cat degradation products in PRR under real processing conditions, comparative experiments between PRR and RRR samples were conducted using liquid chromatography-tandem mass spectrometry (LC-MS) analysis. As a result, **D3** was monitored by the mass spectrometry, while **D1** and **D2** were barely detected in PRR (see Supplementary Materials, Figure S19). Then, an MCI CC method was adopted for the enrichment of degradation products to further verify their unique presence in PRR. Only **D3** was detected from fractions eluted with 60% ethanol in PRR, while none of them were monitored in RRR. As shown in Figure 4(A_1_,A_2_), **D3** was presented exclusively in self-made PRR. This finding provides stronger evidence that steaming induces the production of **D3**, which is absent in the non-steaming-treated samples. To demonstrate the feasibility of **D3** as a specific marker for determining whether RR products have been treated with steaming or not, we detected the enriched-**D3** in six batches of commercially available RRR and PRR samples, respectively. From Figure 4(B_1_,B_2_), **D3** was detected in commercially available PRR with a relatively high content, and it could barely be detected in commercially available RRR. Noteworthily, **D3** was almost not detected in one batch of PRR samples, possibly arising from inadequate processing, which importantly reminds us that the standardized processing cycles and steaming time are necessary to ensure consistent quality in different batches of PRR.

Based on our results, samples containing **D3** were PRR with steaming treatment and **D3** was hardly detected in RRR without steaming, suggesting that **D3** can be served as a potential characteristic marker for differentiating RRR from PRR. However, **D1** and **D2** were not discovered even in enriched PRR samples, which can probably be attributed to the fact that **D3** was produced in great abundance and can be readily available. Moreover, all of them contain carbonyl groups, which can undergo nucleophilic reactions with amino acids to generate more stable nitrogen-containing compounds [28]. Furthermore, intermediate products are likely to undergo further condensation and polymerization reactions to form complex polymer pigments [29].

## 3. Materials and Methods

### 3.1. Reagents and Materials

LC-MS-grade acetonitrile was purchased from Fisher Chemical (Pittsburg, PA, USA). LC-grade methanol and acetonitrile were purchased from Sigma-Aldrich Inc. (St. Louis, MO, USA). LC-grade formic acid was obtained from Shanghai Aladdin Biochemical Technology Co., Ltd. (Shanghai, China). Dimethyl sulfoxide (DMSO) and acetonitrile were both analytical-grade products, provided by Tianjin Damao Chemical Reagent Factory (Tianjin, China). CC was carried out using D101 macroporous resin (Tianjin Chemical Co., Ltd., Tianjin, China) and MCI Gel (CHP20/P120, Mitsubishi Chemical Corporation, Tokyo, Japan). Reference standards including cytidine (Cyt, 18041902), adenosine (Ade, 18041902), guanosine (Gua, 19081902), acteoside (Act, 19082201), and isoacteoside (Iso, 19092702) were acquired from Chengdu Pufei De Biotech Co., Ltd. (Chengdu, China). Cat (1321678), Auc (B21238), RhD (B20293), and Gen (B21661) were obtained from Shanghai Yuanye Biotech Co., Ltd. (Shanghai, China). Leo (Y222002004018) was provided by Chengdu Ruifen Si Biotech Co., Ltd. (Chengdu, China). The purity of all standards was above 98% following liquid chromatography–ultraviolet (LC-UV) analysis. Yellow wine was purchased from Zhejiang Guyuelongshan Shaoxing Wine Co., Ltd. (Zhejiang, China).

Three batches of FRR samples were collected from Jiaozuo city, Henan province of China, in November 2020. Nine batches of RRR and six batches of PRR were purchased from Hebei Chunkai Pharmaceutical Co., Ltd. (Hebei, China) and were all authenticated by Prof. Tianxiang Li of Tianjin University of Traditional Chinese Medicine (Tianjin, China).

### 3.2. Preparation of RRR and PRR

To acquire RRR, three batches (2500 g/batch) of FRR with a uniform size (weight of 110 ± 15 g, diameter of 58 ± 10 mm) were selected, with their fibrous roots removed. The FRR samples were then cut and dried at 50 °C for 10 days (10 h per day), and they were collected on days 2, 4, 6, 8, and 10 marked as RRR-2, RRR-4, RRR-6, RRR-8, and RRR-10, respectively.

PRRs were obtained through the following procedure: RRR-10 samples were picked and mixed with yellow wine at a weight ratio of 10:4, followed by steaming for 12 h until softened. The softened samples were then blended with steaming liquid, steamed for another 6 h, and then dried for 6 h at 60 °C until the texture became viscous and dark-black colored. Samples were finally cut into homogeneous pieces (about 2~4 mm) and dried for use. Moreover, three batches of commercially available RRR samples were randomly selected and then processed to PRR, named PRR-1, PRR-2, and PRR-3, using the above method.

### 3.3. Preparation of Standard and Sample Solutions

#### 3.3.1. Preparation of Standard and Sample Solutions for Quantitative Analysis

Ten reference standards were accurately weighed and dissolved in methanol to obtain stock solutions separately, which were then used to prepare a mixed standard solution with final concentrations of 4.016 µg/mL Cyt, 3402 µg/mL Cat, 5.030 µg/mL Ade, 1.761 µg/mL Gua, 48.42 µg/mL Auc, 242.4 µg/mL RhD, 240.2 µg/mL Leo, 2.515 µg/mL Gen, 12.75 µg/mL Act, and 3.006 µg/mL Iso. Subsequently, the mixed standard solution was serially diluted with 50% methanol aqueous solution to obtain seven different concentrations for establishing the calibration curves.

Accurately weighed sample powder (0.5 g) was ultrasonically extracted (144 W, 135 kHz) with 20 mL 35% methanol aqueous solution at 30 ℃ for 50 min. Then, 35% methanol aqueous solution was added to compensate for weight lost during extraction. After centrifugation at 13,322 g for 10 min, the supernatant was collected and filtered through a 0.22 µm membrane filter, which was then diluted with ultrapure water (*v:v*, 1:1) to obtain the sample solution.

#### 3.3.2. Preparation of Cat Standard Solution for Identification of Degradation Products

The acetate buffer solution at pH 4.8 was prepared by mixing 8.0 mL acetic acid and 9.0 g sodium acetate anhydrous dissolved in 500 mL ultrapure water. A stock solution of Cat was prepared at 3.620 mg/mL in acetate buffer solution. Afterwards, 1 mL stock solution was mixed with 9 mL buffer solution in centrifuge tube and incubated for 48 h at 100 °C.

#### 3.3.3. Preparation of Sample Solutions for the Validation of Cat Degradation Products

About 5.0 g RRR/PRR powder was ultrasonically extracted with 200 mL of 50% methanol aqueous solution for 50 min. The filtrate (115 mL) was accurately measured and evaporated under reduced pressure to obtain 10 mL concentrated solution for isolation.

The concentrated solution was chromatographed on an MCI column, which was eluted with EtOH-H_2_O (30:70, 50:50, 70:30, *v*/*v*) to afford fractions with Cat degradation products. Each fraction was then concentrated using a rotary vacuum evaporator (EYELA, Tokyo, Japan) and diluted to 3 mL with methanol for LC-MS and UPLC-PDA analysis.

### 3.4. UPLC-PDA Conditions

An ACQUITY UPLC I-class system (Waters Corporation, Milford, MA, USA) was used to perform the chromatographic analysis by using ACQUITY UPLC^®^ HSS T3 column (2.1 × 100 mm, 1.8 µm) at 35 °C for the quantitative analysis. The mobile phase was composed of 0.1% formic acid aqueous solution (A) and acetonitrile (B) and then implemented in the gradient elution as follows: 0–1 min, 0–1% B; 1–3 min, 1–5% B; 3–4 min, 5–5% B; 4–6 min, 5–8% B; 6–13 min, 8–17% B; 13–20 min, 17–22% B.

A Waters ACQUITY UPLC^®^ BEH C18 (2.1 × 100 mm, 1.7 µm) maintained at 55 °C was used for the identification of Cat degradation products. The mobile phase consisted of 0.1% formic acid solution (A) and acetonitrile (B) using the following gradient program: 0–20 min, 1–95% B; 20–21 min, 95–1% B. Both of them were delivered at flow rate of 0.3 mL/min, and the sample injection was 2 µL. The detection wavelength was set at 203 and 254 nm.

### 3.5. Methodological Validation

According to the guidelines released by ChP [2], the analytical method established in this study was employed to validate linearity, LOD, LOQ, precision (intra- and inter-day), stability, repeatability, and a recovery test. The calibration curves were drawn using the peak area (*y*-axis) and the corresponding concentrations of the compounds (*x*-axis). The LOQ and LOD of the tested compounds were determined by gradually reducing the concentration of the standard solution until the signal-to-noise ratio (S/N) was about 10 and 3, respectively. In order to test the intra- and inter-day precisions, the sample solution was injected six times on the same day and for three consecutive days, subsequently. Repeatability was confirmed by preparing and analyzing six sample solutions. The stability was studied by injecting sample solution at 0, 2, 4, 6, 8, 10, 12, and 24 h. The recovery test was carried out by adding standard solution to 0.25 g sample powder (*n* = 6), which was processed according to the method of sample preparation.

### 3.6. Purification and Enrichment of Degradation Products of Cat

#### 3.6.1. Isolation of Degradation Samples

Accurately weighed 905.8 mg of Cat was dissolved in pH 4.8 buffer solution to obtain 5 mM stock solution, which was incubated at 100 °C for 12 h. After cooling, the solution was evaporated to gain concentrated Cat reaction solution, which was subsequently loaded onto a D101 macroporous resin column and eluted with various ratios of EtOH-H_2_O solutions (0:100, 30:70, 50:50, 70:30, *v:v*) to give four fractions (Frs. 1–4).

#### 3.6.2. Purification by Preparative HPLC

Fr. 2 was purified on an Agilent 1260 Infinity preparative HPLC system, which was equipped with Waters SunFire^®^ C18 OBDTM Prep Column (10 × 250 mm, 5 µm). Then, 0.1% formic acid aqueous solution–acetonitrile was used as the mobile phase with isocratic elution (91:9, *v*/*v*) at a flow rate of 4.0 mL/min, and the column temperature was maintained at room temperature. The injection volume was 100 µL and the detector was set at 203 and 254 nm to obtain **D1**. The purification of Fr. 3 was chromatographed in an isocratic elution of 0.1% formic acid aqueous solution–methanol (65:35, *v*/*v*) to give **D2**. Fr. 4 was also chromatographed in an isocratic elution of 0.1% formic acid aqueous solution–methanol (55:45, *v*/*v*) to yield **D3**. The final products were subjected to NMR analysis for structural elucidation.

### 3.7. NMR Spectroscopic Analysis

For NMR analysis, we used deuterated dimethylsulfoxide (DMSO-*d*_6_) and methanol-*d*_4_ as the solvents, and tetramethylsilane (TMS) as the internal standard. Then, 1D (^1^H NMR and ^13^C NMR) and 2D (^1^H-^1^H COSY, HSQC, and HMBC) NMR experiments were performed on a Bruker AVIII 600 spectrometer (Bruker, Zug, Switzerland). The chemical shifts (*δ*) are in ppm and the coupling constants (*J*) are in Hz.

### 3.8. LC-MS Analysis

The chromatographic separation was performed on Vanquish UHPLC System (Thermo Fisher Scientific, San Jose, CA, USA) with an ACQUITY UPLC^®^ HSS T3 column (2.1 × 100 mm, 1.8 µm) maintained at 55 °C. Mobile phases consisted of 0.1% formic acid aqueous solution (A) and acetonitrile (B), with the following gradient elution procedure: 0–15 min, 5–27% B; 15–16 min, 27–95% B. The flow rate of elution solvent was 0.3 mL/min and the injection volume of samples was 2 µL. UV detection wavelength was set at 203 and 254 nm.

The mass spectrometry analysis was performed on an Orbitrap Exploris 120 Mass Spectrometer (Thermo Fisher Scientific). The ESI source parameters were set as follows: spray voltage, −3.0 kV/+3.5 kV; ion transfer tube temperature, 320 °C; vaporizer temperature, 350 °C; normalized collision energy, 20/40/60 V; sheath gas (N_2_), 35 arb; aux gas (N_2_), 10 arb; sweep gas (N_2_), 0 arb. Moreover, the scanning method of full MS was adopted. The full scan range of MS^1^ was 50–1500 *m*/*z* acquired with resolution R = 120,000.

### 3.9. Data Analysis

The data were presented as mean ± SD and analyzed using one-way ANOVA by SPSS 19.0 (IBM, Armonk, NY, USA). The difference was considered to be statistically significant if *p* < 0.05. Graphs were created using Origin 9.1 (Originlab Corp., Northampton, MA, USA). The RSD value reflects the precision of the analytical results in the assay.

## 4. Conclusions

In this study, the UPLC-PDA method for the quantitative analysis of iridoid glycosides, nucleosides, and phenylethanoid glycosides was successfully established and applied in clarifying the dynamic variations of compounds during the process from FRR to PRR. A sharp decrease in iridoid glycosides was noticed in content from RRR to PRR, which was successfully simulated under processing conditions by employing Cat as an example. From Cat, jiofuraldehyde, cataldehyde, and norviburtinal were obtained and elucidated as the degradation products and cataldehyde was reported as a new compound. By unveiling the specificity of norviburtinal in PRR, the RRR samples can be easily distinguished from the PRR. Overall, our study further revealed the processing mechanism of RR, providing insights into the transformation of effective constituents. However, it still leaves a large space for us to explore the relationship between Cat degradation products and the changes in the pharmacological effects of different RR products.

## Figures and Tables

**Table 1:** Methodological validation for simultaneous quantification of ten compounds in different RR products.

Compounds	Linear Regression			LODs	LOQs	Precision (RSD,%)		Repeatability	Stability	Recovery
	Regression Equation	*r* ^2^	Linear Range (μg/mL)	(μg/mL)	(μg/mL)	Intra-Day (*n* = 6)	Inter-Day (*n* = 3)	(*n* = 6, RSD, %)	(*n* = 7, RSD, %)	(*n* = 6, Mean ± SD, %)
Cyt	*y* = 4204.9*x* − 297.17	0.9997	0.0992–2.008	0.0331	0.0992	2.0	1.9	0.6	2.3	101.58 ± 1.7
Cat	*y* = 1326.1*x* + 7233.5	0.9996	84.00–1701	9.3334	28.000	1.3	1.0	0.9	0.8	98.30 ± 3.0
Ade	*y* = 22932*x* + 324.90	1.0000	0.1242–2.515	0.0138	0.0414	2.6	2.9	2.0	2.8	98.31 ± 3.6
Gua	*y* = 18807*x* + 203.55	0.9996	0.04347–0.8803	0.0145	0.0435	2.0	2.7	1.5	2.1	96.66 ± 1.0
Auc	*y* = 16688*x* − 2154.9	0.9996	1.196–24.21	0.3985	1.1956	2.3	2.8	2.4	2.5	99.36 ± 3.3
RhD	*y* = 5738.1*x* + 19552	0.9993	5.985–121.2	1.9951	5.9852	1.1	2.3	2.8	2.7	99.90 ± 3.2
Leo	*y* = 8574.4*x* + 29466	0.9990	5.931–120.1	0.6590	1.9770	0.8	2.2	1.5	1.6	92.87 ± 4.5
Gen	*y* = 13584*x* − 8.9794	0.9999	0.06210–1.258	0.0207	0.0621	2.6	2.4	1.8	2.0	91.49 ± 2.8
Act	*y* = 13794*x* + 307.19	1.0000	0.3148–6.375	0.0350	0.1049	2.1	3.0	2.9	1.8	93.41 ± 4.9
Iso	*y* = 13299*x* + 431.85	0.9996	0.07422–1.503	0.0247	0.0742	–	–	–	–	96.92 ± 1.2

Abbreviation notes: Cyt: cytidine; Cat: catalpol; Ade: adenosine; Gua: guanosine; Auc: aucubin; RhD: rehmannioside D; Leo: leonuride; Gen: geniposide; Act: acteoside; Iso: isoacteoside.

**Table 2:** ^1^H NMR (600 MHz) and ^13^C NMR (150 MHz) spectroscopic data of compounds **D1**–**D3**.

Position	D1 (in DMSO-*d*_6_)		D2 (in CD_3_OD)		D3 (in CD_3_OD)	
	*δ*_H_	*δ*_C_	*δ*_H_	*δ*_C_	*δ*_H_	*δ*_C_
2	7.52 (1H, s)	136.0	–	157.5	8.11 (1H, d, 4.8)	146.5
3	–	130.5	7.27 (1H, s)	115.0	7.56 (1H, d, 4.8)	112.3
4	4.60 (1H, d, 4.8)	65.9	–	137.9	–	139.0
5	3.79 (1H, dd, 4.8, 8.4)	83.2	7.33 (1H, brs)	123.1	6.71 (1H, d, 3.6)	112.7
6	3.15 (1H, m)	36.3	7.33 (1H, s)	133.4	8.09 (1H, d, 3.6)	146.5
7	–	130.2	–	133.2	–	123.2
8	7.17 (1H, s)	134.3	2.98 (1H, dd, 5.4, 13.8) 2.90 (1H, dd, 5.4, 13.8)	39.6	–	124.0
9	2.60 (1H, ddd, 1.8, 9.0, 17.4) 2.85 (1H, ddd, 1.8, 5.4, 17.4)	45.8	4.85 (1H, m, overlapped)	98.7	9.27 (1H, s)	152.6
10	9.82 (1H, t, 1.8)	202.9	9.85 (1H, s)	194.0	9.66 (1H, s)	187.3

## Data Availability

Data are contained within the article and Supplementary Materials.

## Supplementary Materials

The following supporting information can be downloaded at https://www.mdpi.com/article/10.3390/molecules29030705/s1, Table S1: Optimization of the extracting method for the sample preparation of RR; Figure S1: ^1^H NMR spectrum of **D1** in DMSO-*d*_6_ (600 MHz); Figure S2: ^13^C NMR spectrum of **D1** in DMSO-*d*_6_ (150 MHz); Figure S3: ^1^H-^1^H COSY spectrum of **D1** in DMSO-*d*_6_; Figure S4: HSQC spectrum of **D1** in DMSO-*d*_6_; Figure S5: HMBC spectrum of **D1** in DMSO-*d*_6_; Figure S6: HR-ESI-MS spectrum of **D1**; Figure S7: ^1^H NMR spectrum of **D2** in CD_3_OD (600 MHz); Figure S8: ^13^C NMR spectrum of **D2** in CD_3_OD (150 MHz); Figure S9: ^1^H-^1^H COSY spectrum of **D2** in CD_3_OD; Figure S10: HSQC spectrum of **D2** in CD_3_OD; Figure S11: HMBC spectrum of **D2** in CD_3_OD; Figure S12: HR-ESI-MS spectrum of **D2**; Figure S13: ^1^H NMR spectrum of **D3** in CD_3_OD (600 MHz); Figure S14: ^13^C NMR spectrum of **D3** in CD_3_OD (150 MHz); Figure S15: ^1^H-^1^H COSY spectrum of **D3** in CD_3_OD; Figure S16: HSQC spectrum of **D3** in CD_3_OD; Figure S17: HMBC spectrum of **D3** in CD_3_OD; Figure S18: HR-ESI-MS spectrum of **D3**; Figure S19. Representative total ion current (TIC) chromatogram of PRR sample in the positive ion mode (A) and MS spectrum of **D3** in PRR sample (B).

## Abbreviations

RR	Radix Rehmanniae
*R. glutinosa*	*Rehmannia glutinosa* Libosch.
TCM	traditional Chinese medicine
FRR	fresh Radix Rehmanniae
RRR	raw Radix Rehmanniae
PRR	processed Radix Rehmanniae
Cat	catalpol
Leo	leonuride
RhD	rehmannioside D
ChP	Chinese pharmacopoeia
UPLC-PDA	ultra-performance liquid chromatography with photo-diode array detector
LOD	the limit of detection
LOQ	the limit of quantitation
RSD	relative standard deviation
Auc	aucubin
Gen	geniposide
CC	column chromatography
preparative HPLC	preparative high-performance liquid chromatography
NMR	nuclear magnetic resonance
HMBC	heteronuclear multiple bond correlation
^1^H-^1^H COSY	^1^H-^1^H correlated spectroscopy
LC-MS	liquid chromatography–tandem mass spectrometry
DMSO	dimethyl sulfoxide
Cyt	cytidine
Ade	adenosine
Gua	guanosine
Act	acteoside
Iso	isoacteoside
LC-UV	liquid chromatography–ultraviolet
S/N	signal-to-noise ratio
TMS	tetramethylsilane
