# Supplementary material for: Exploration of the Dynamic Variations of the Characteristic Constituents and the Degradation Products of Catalpol during the Process of Radix Rehmanniae
Source: Molecules. 2024 Feb 3;29(3):705. doi: 10.3390/molecules29030705 (PMC10856693; doi:10.3390/molecules29030705)
Supplement: Supplementary file 1 [file molecules-29-00705-s001.zip › molecules-2832220-supplementary.pdf]

Supplementary Materials

Exploration on the Dynamic Variations of the Characteristic Constituents and the Degradation Products of Catalpol during the Process of Radix Rehmanniae

Jingjing Yang [1,†], Lihua Zhang [1,†], Mengyue Zhang [1], Mingxuan Yang [1], Lin Zou [1], Ying Cui [1], Jing Yang [1], Xin Chai [1,2,*] and Yuefei Wang [1,2,*]

[1] National Key Laboratory of Chinese Medicine Modernization, State Key Laboratory of Component-based Chinese Medicine, Tianjin Key Laboratory of TCM Chemistry and Analysis,
Tianjin University of Traditional Chinese Medicine, Tianjin 301617, China; yangpgjj@163.com (J.Y.);
16622765055@163.com (L.Z.); zhangmengyue1113@163.com (M.Z.); 15093240403@163.com (M.Y.);
linzou230505@126.com (L.Z.); cql8179270@tjutcm.edu.cn (Y.C.); yangjingoffice@163.com (J.Y.)

[2] Haihe Laboratory of Modern Chinese Medicine, Tianjin 301617, China

* Correspondence: chaix0622@tjutcm.edu.cn (X.C.); wangyf0622@tjutcm.edu.cn (Y.W.); Tel.: +86-22-5959-6366 (X.C. & Y.W.)

† These authors contributed equally to this work.

Table of Contents

Table S1. Optimization of the extracting method for the sample preparation of RR

Figure S1. ^{1}H NMR spectrum of D**1** in DMSO-d_6 (600 MHz)

Figure S2. ^{13}C NMR spectrum of D**1** in DMSO-d_6 (150 MHz)

Figure S3. ^{1}H-^{1}H COSY spectrum of D**1** in DMSO-d_6

Figure S4. HSQC spectrum of D**1** in DMSO-d_6

Figure S5. HMBC spectrum of D**1** in DMSO-d_6

Figure S6. HR-ESI-MS spectrum of D**1**

Figure S7. ^{1}H NMR spectrum of D**2** in CD_3OD (600 MHz)

Figure S8. ^{13}C NMR spectrum of D**2** in CD_3OD (150 MHz)

Figure S9. ^{1}H-^{1}H COSY spectrum of D**2** in CD_3OD

Figure S10. HSQC spectrum of D**2** in CD_3OD

Figure S11. HMBC spectrum of D**2** in CD_3OD

Figure S12. HR-ESI-MS spectrum of D**2**

Figure S13. ^{1}H NMR spectrum of D**3** in CD_3OD (600 MHz)

Figure S14. ^{13}C NMR spectrum of D**3** in CD_3OD (150 MHz)

Figure S15. ^{1}H-^{1}H COSY spectrum of D**3** in CD_3OD

Figure S16. HSQC spectrum of D**3** in CD_3OD

Figure S17. HMBC spectrum of D**3** in CD_3OD

Figure S18. HR-ESI-MS spectrum of D**3**

Figure S19. Representative total ion current (TIC) chromatogram of PRR sample in the positive ion mode (A) and MS spectrum of D3 in PRR sample (B)

Table S1. Optimization of the extracting method for the sample preparation of RR

Elements affecting the extraction efficiency of constituents in RR			
Extraction solvent	Extraction temperature （°C）	Material/solvent ratio (g:mL)	Ultrasonic time (min)
30% methanol aqueous	room temperature	1:80	20
35% methanol aqueous	30	1:40	30
40% methanol aqueous	40	1:20	40
45% methanol aqueous	50	—	50
50% methanol aqueous	60	—	60
75% methanol aqueous	—	—	—
methanol	—	—	—

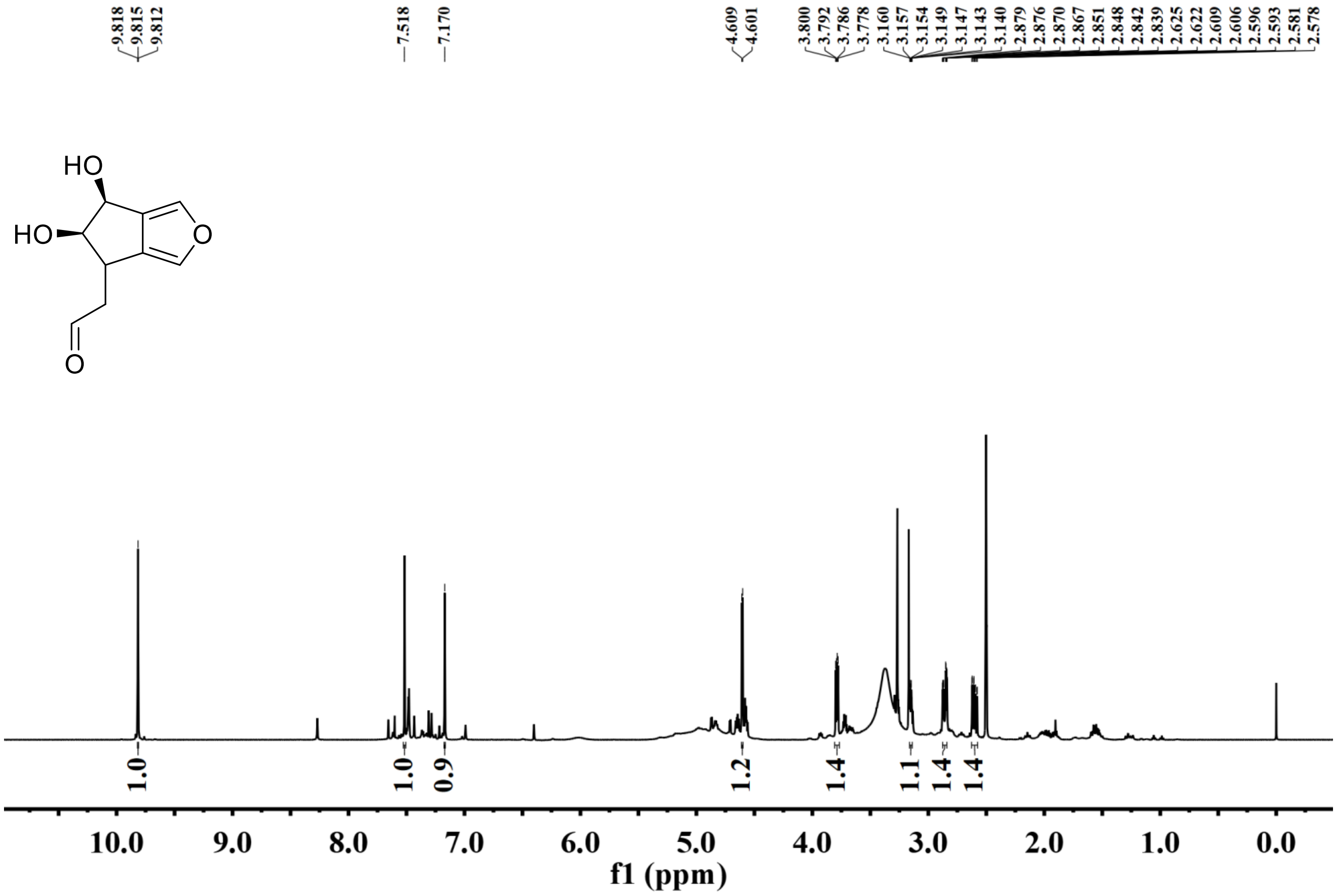

Figure S1. ^{1}H NMR spectrum of D**1** in DMSO-d_6 (600 MHz).

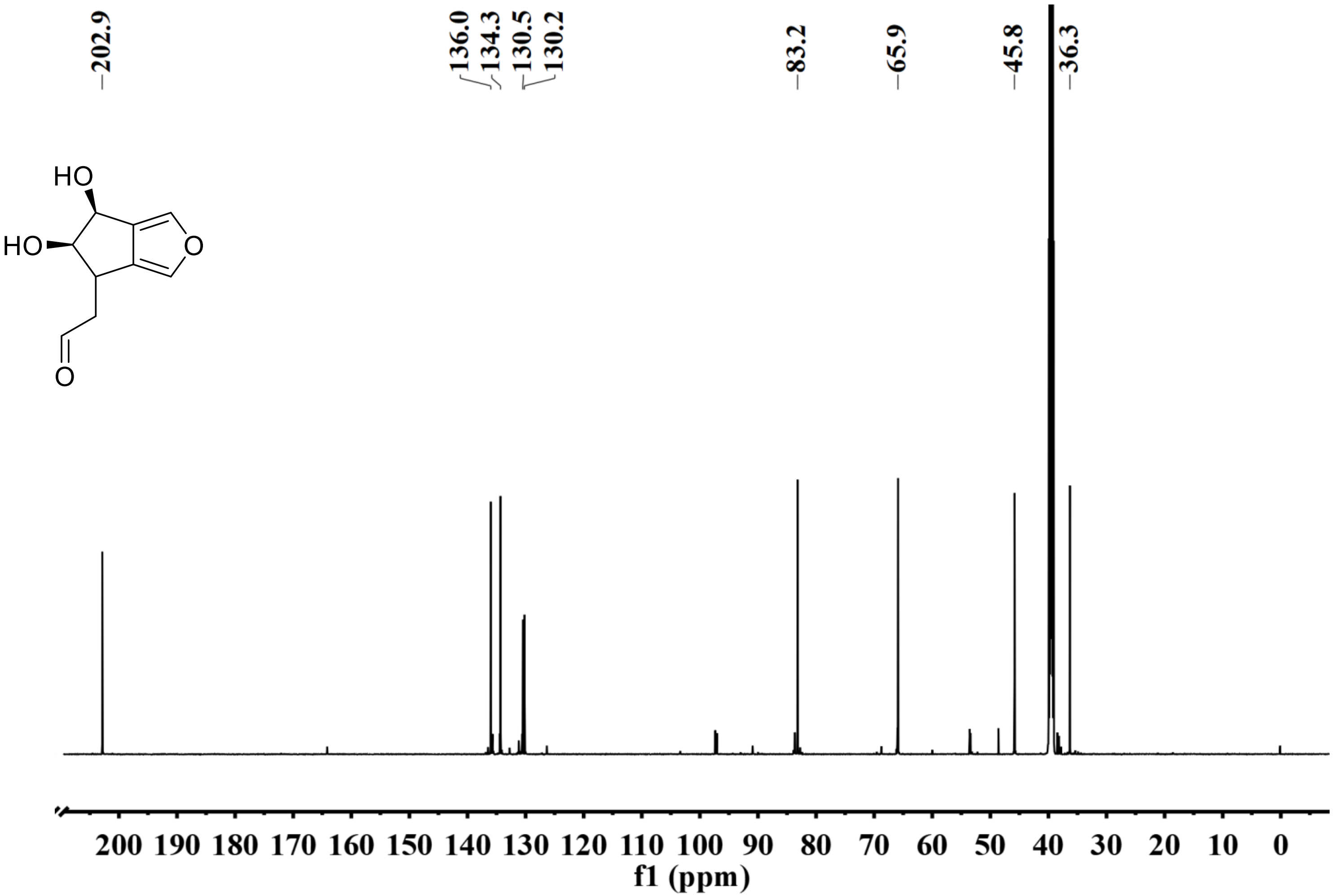

Figure S2. ^{13}C NMR spectrum of D**1** in DMSO-d_6 (150 MHz)

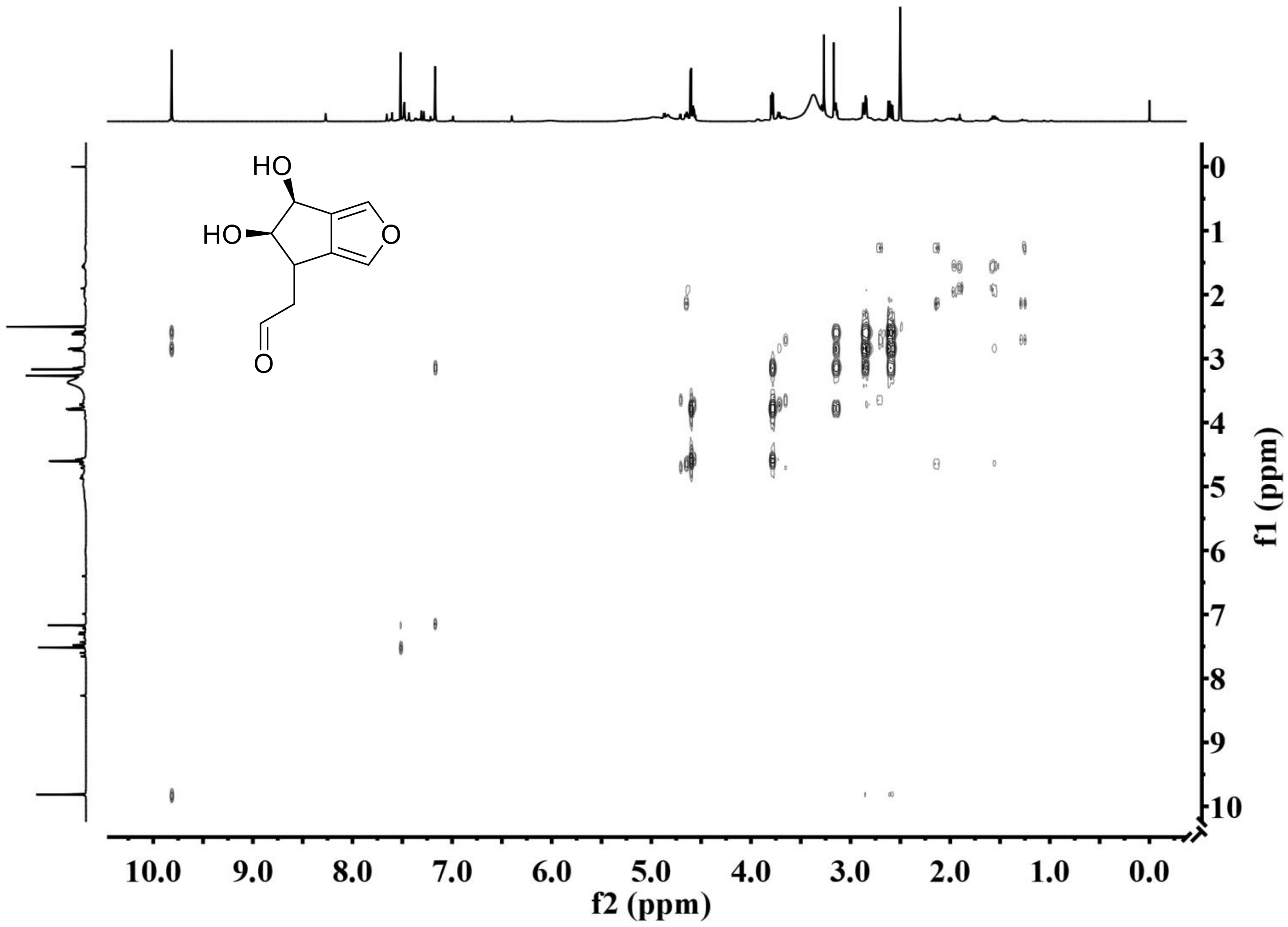

Figure S3. ^1H-^1H COSY spectrum of D**1** in DMSO-d_6

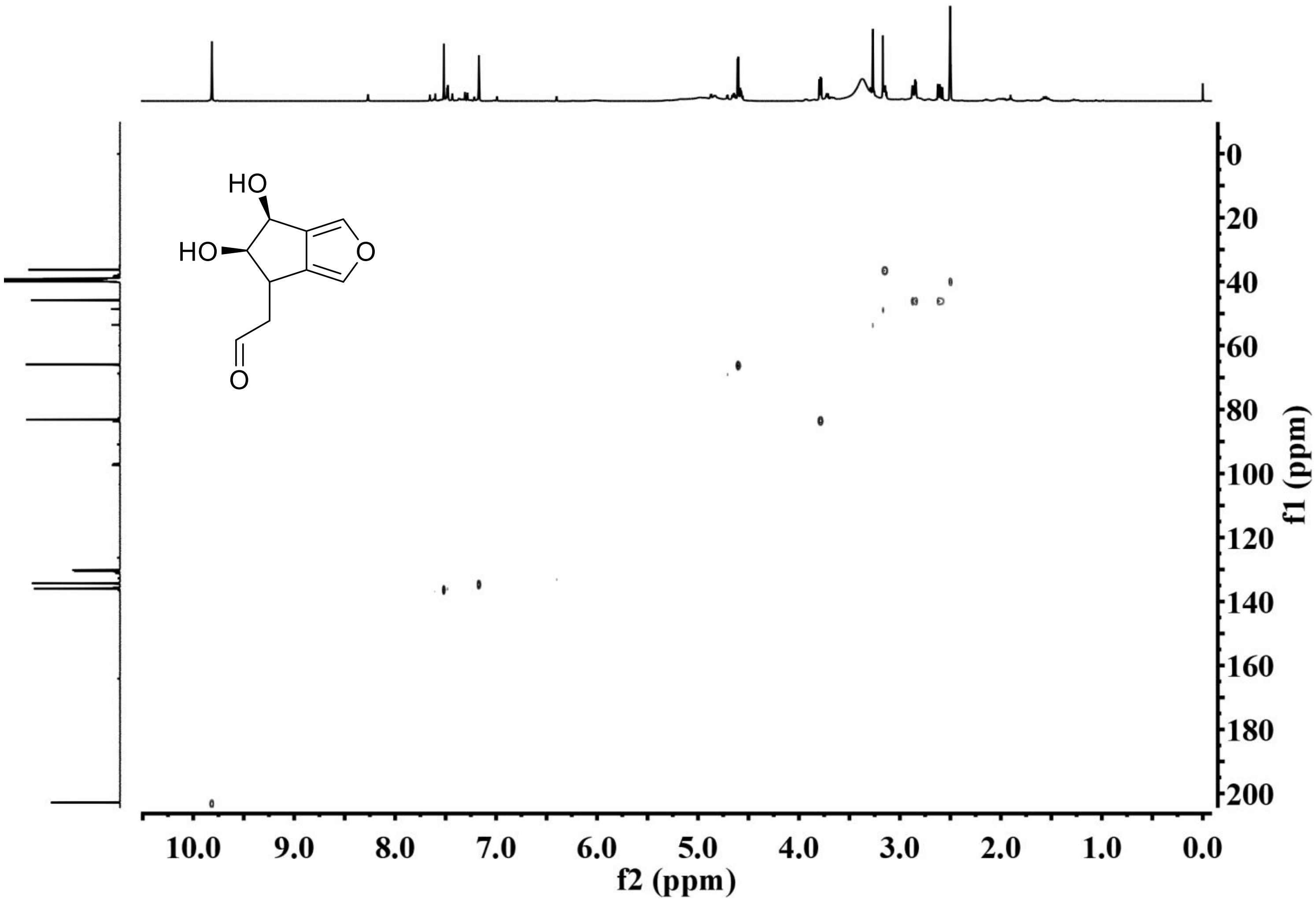

Figure S4. HSQC spectrum of D**1** in DMSO-d_6

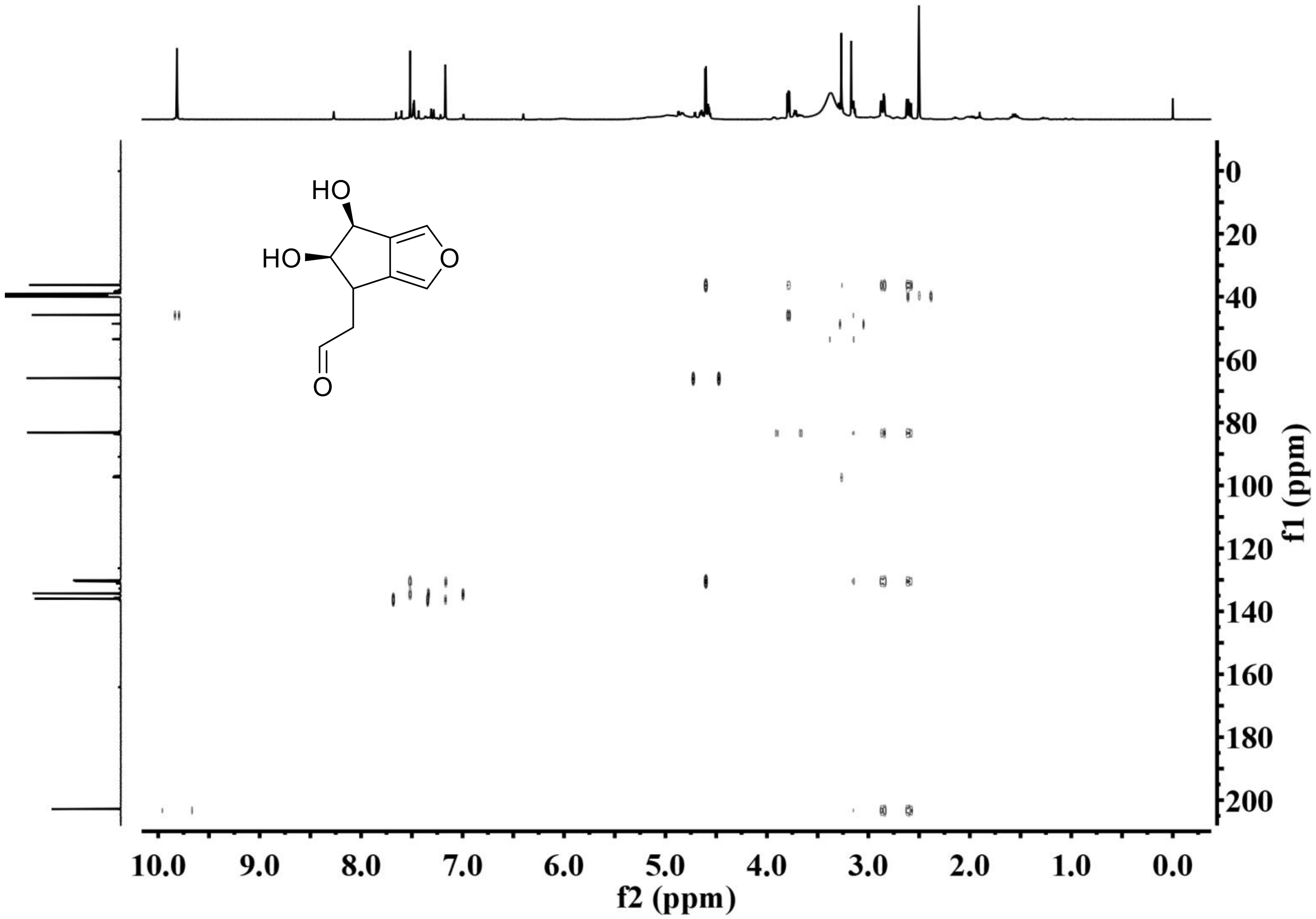

Figure S5. HMBC spectrum of D**1** in DMSO-d_6

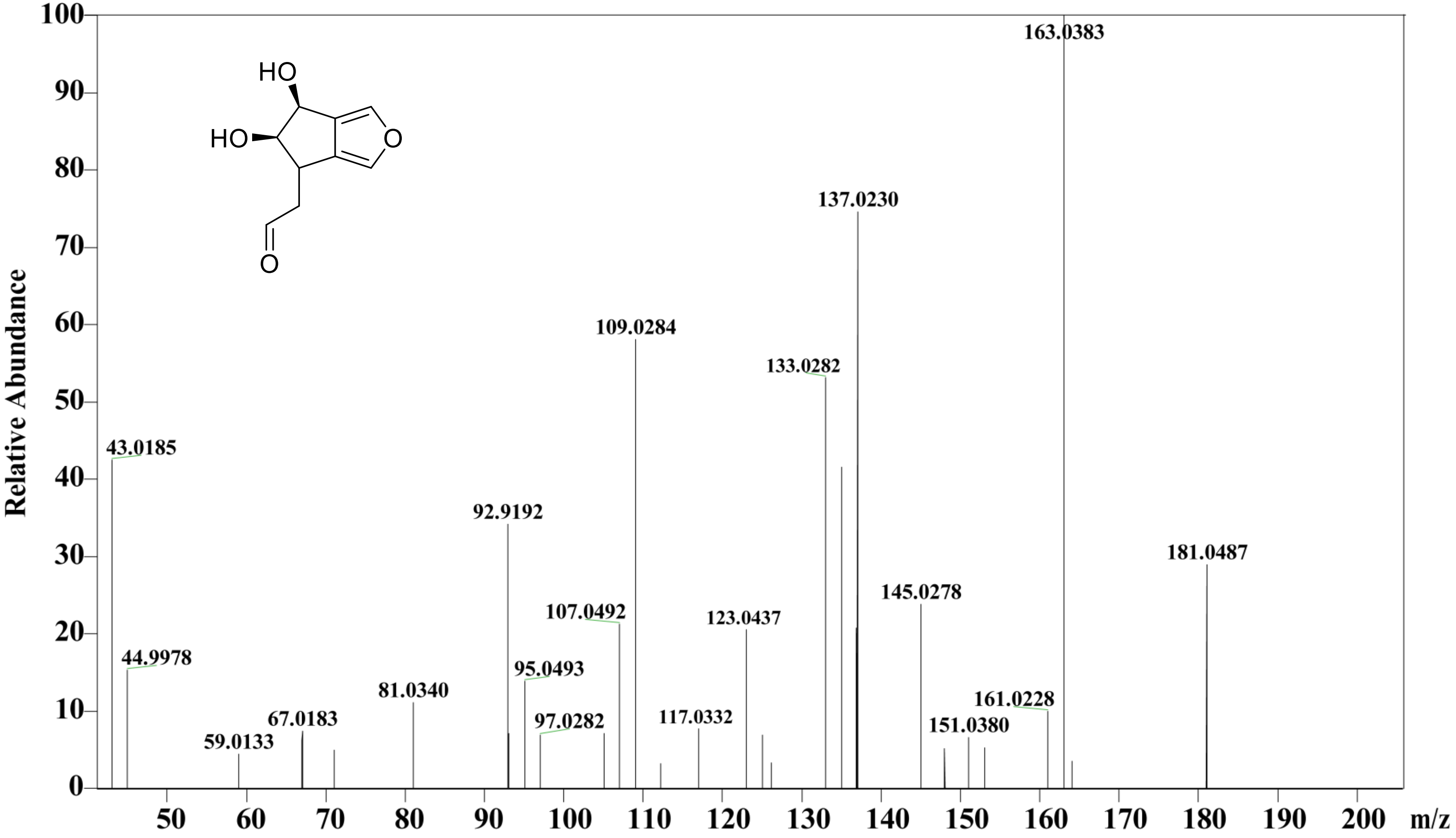

Figure S6. HR-ESI-MS spectrum of D**1**

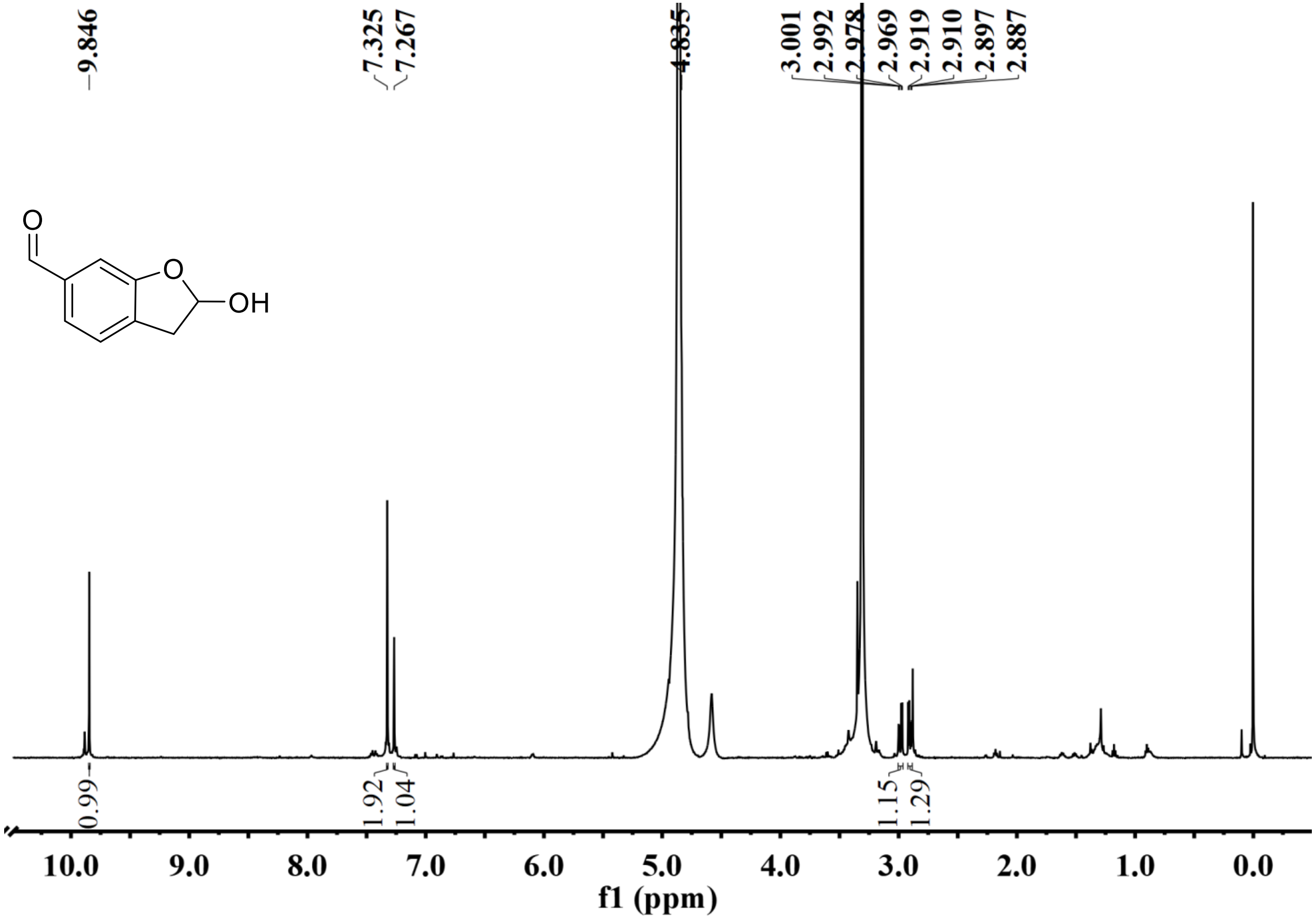

Figure S7. ^{1}H NMR spectrum of D**2** in CD_3OD (600 MHz)

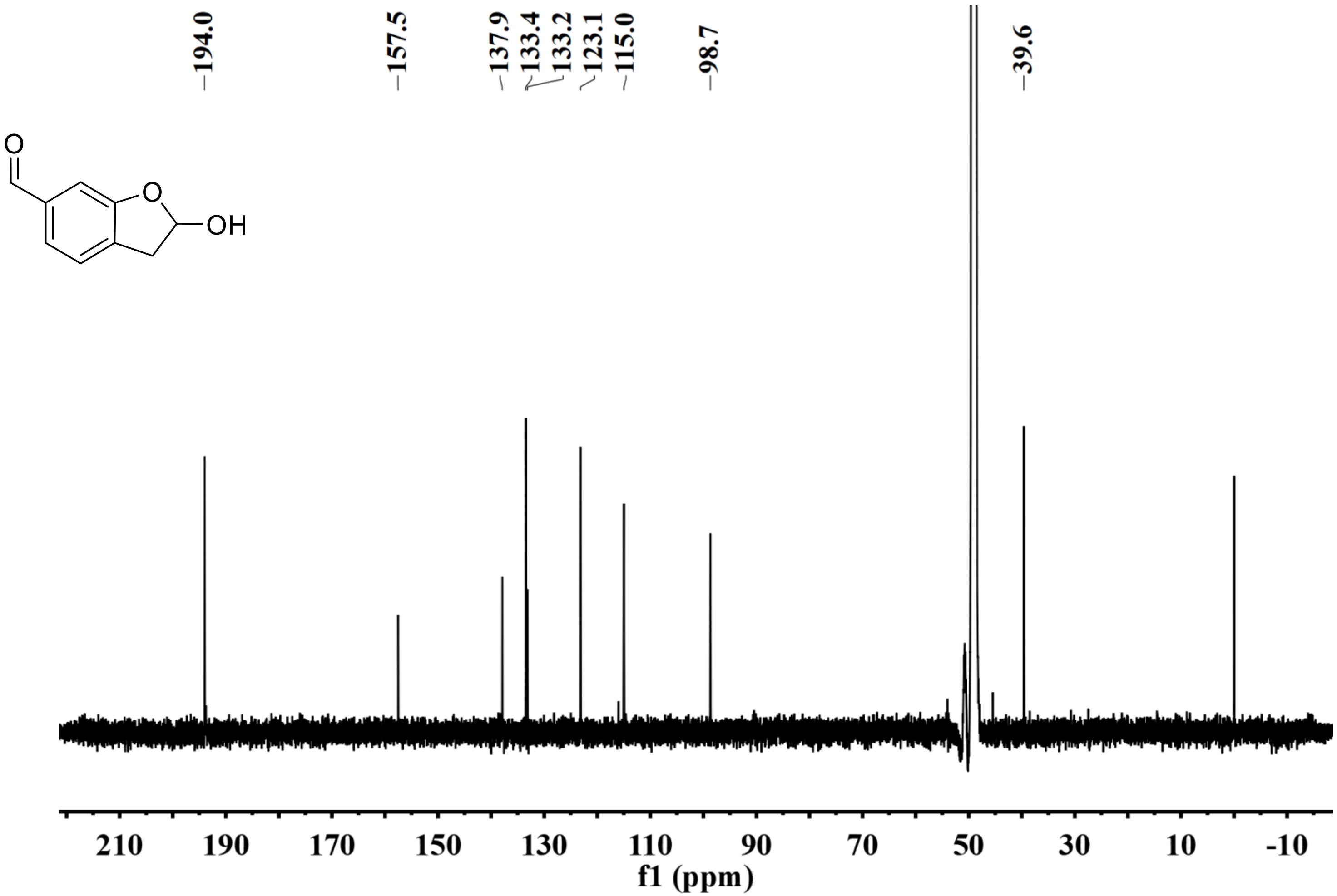

Figure S8. ^{13}C NMR spectrum of D**2** in CD_3OD (150 MHz)

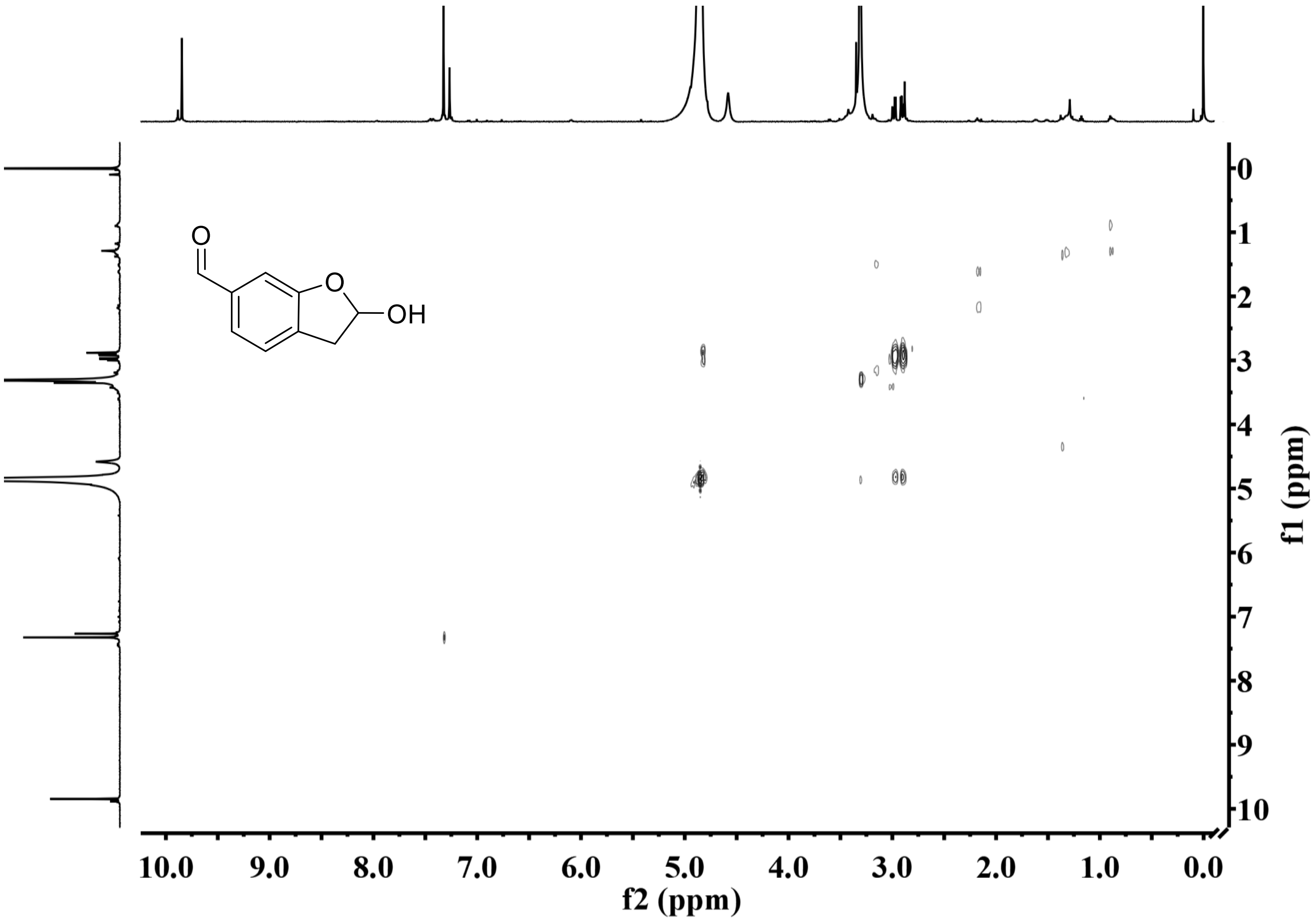

Figure S9. ^{1}H-^{1}H COSY spectrum of D**2** in CD_3OD

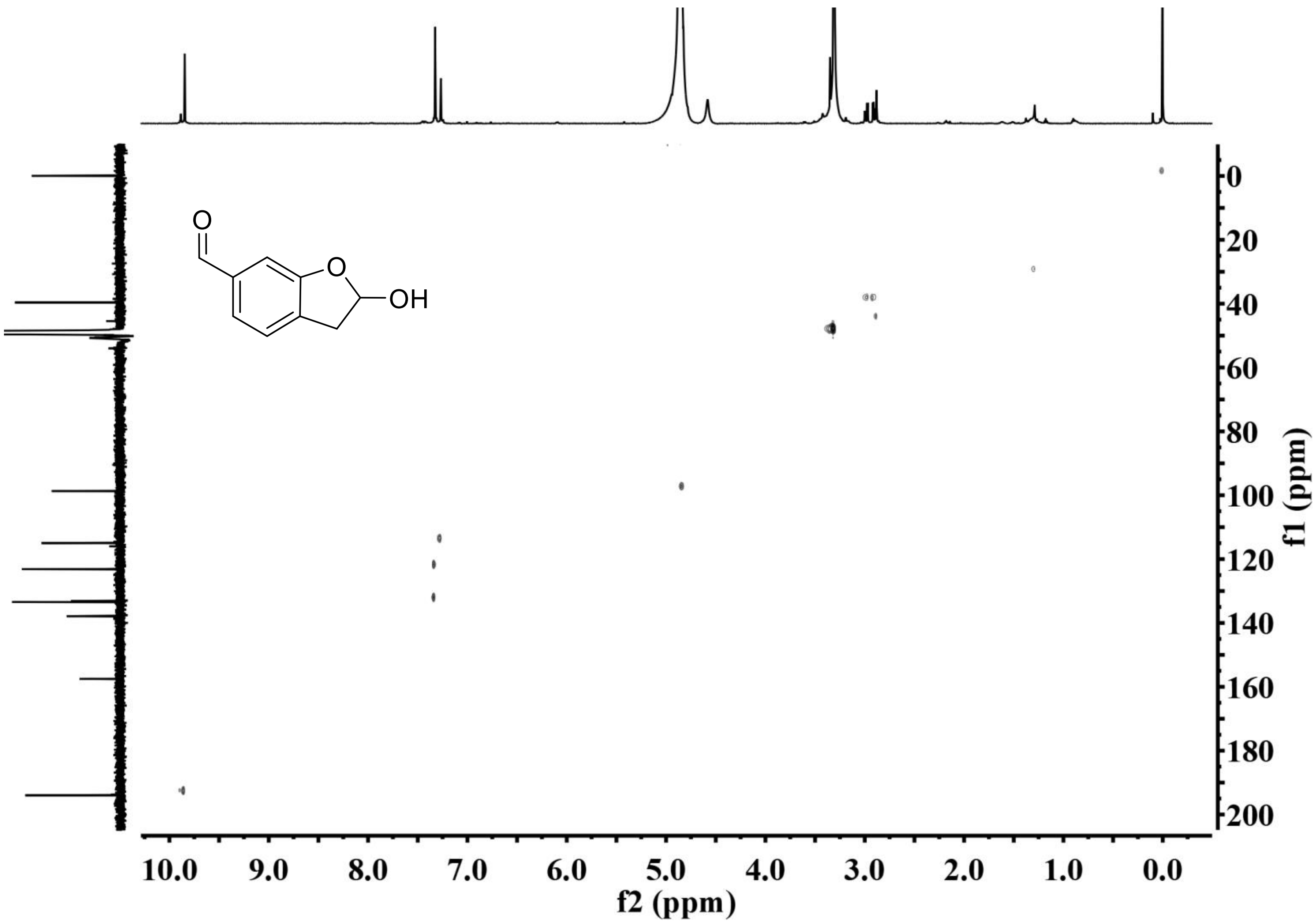

Figure S10. HSQC spectrum of D**2** in CD_3OD

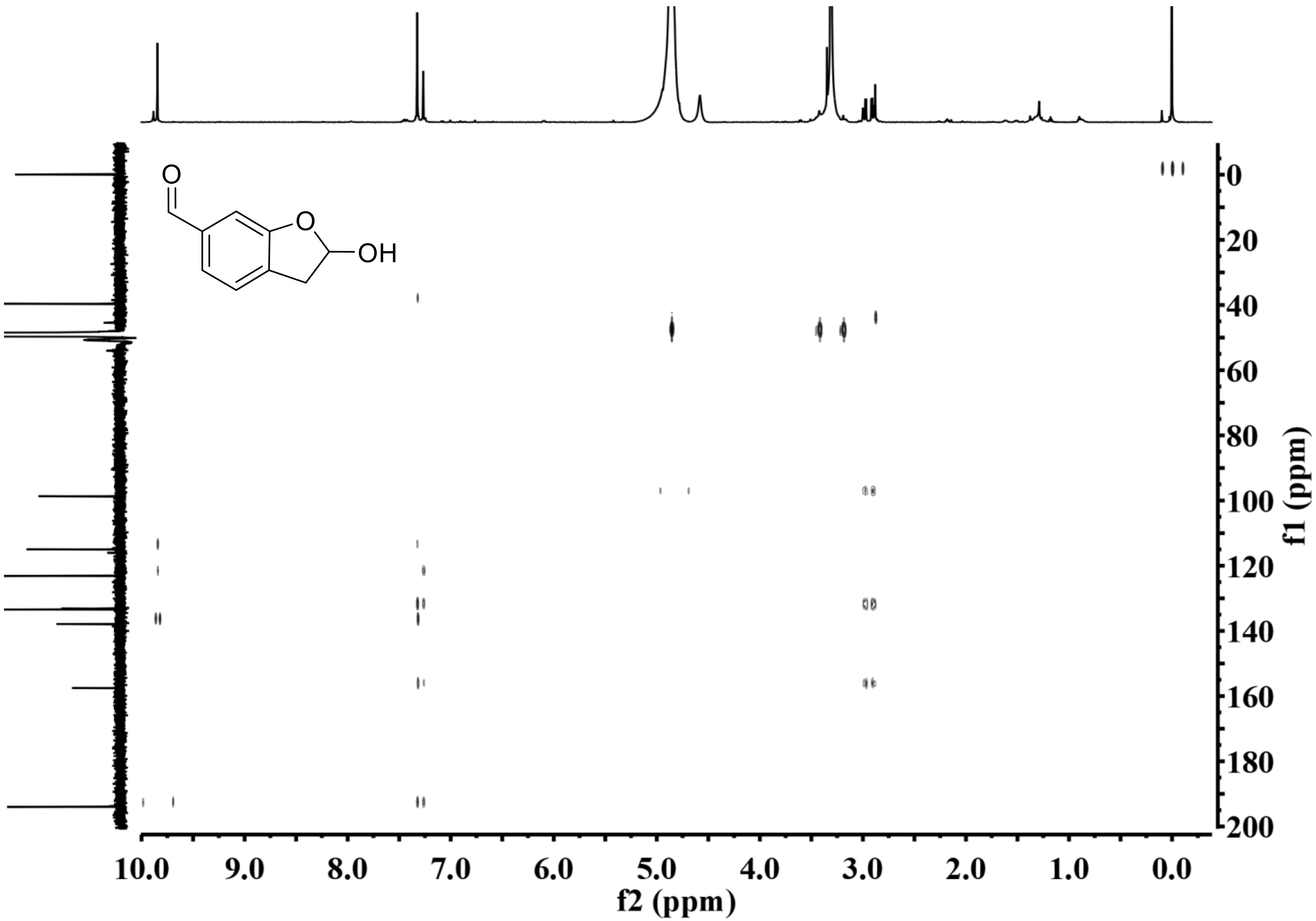

Figure S11. HMBC spectrum of D**2** in CD_3OD

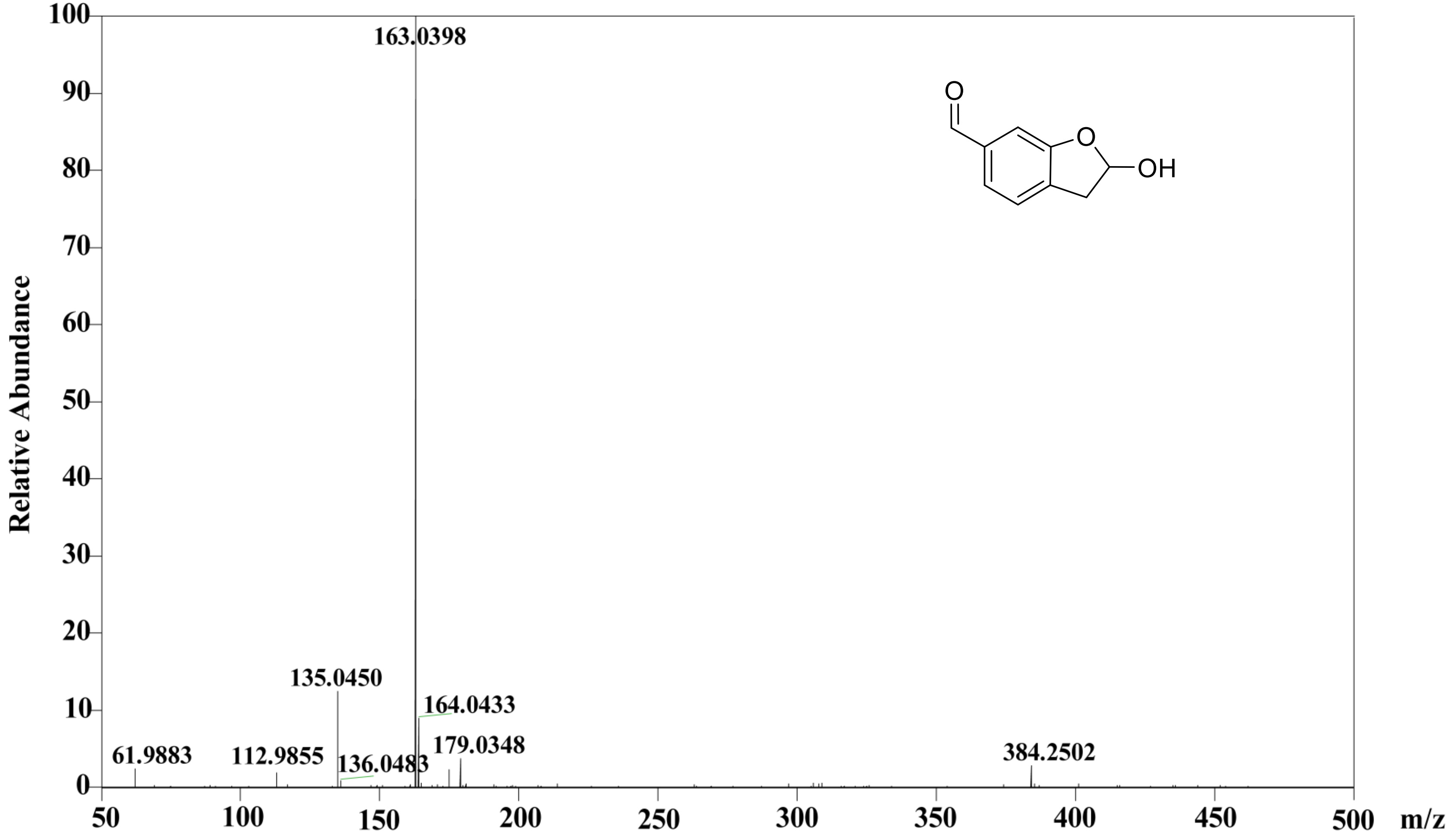

Figure S12. HR-ESI-MS spectrum of D**2**

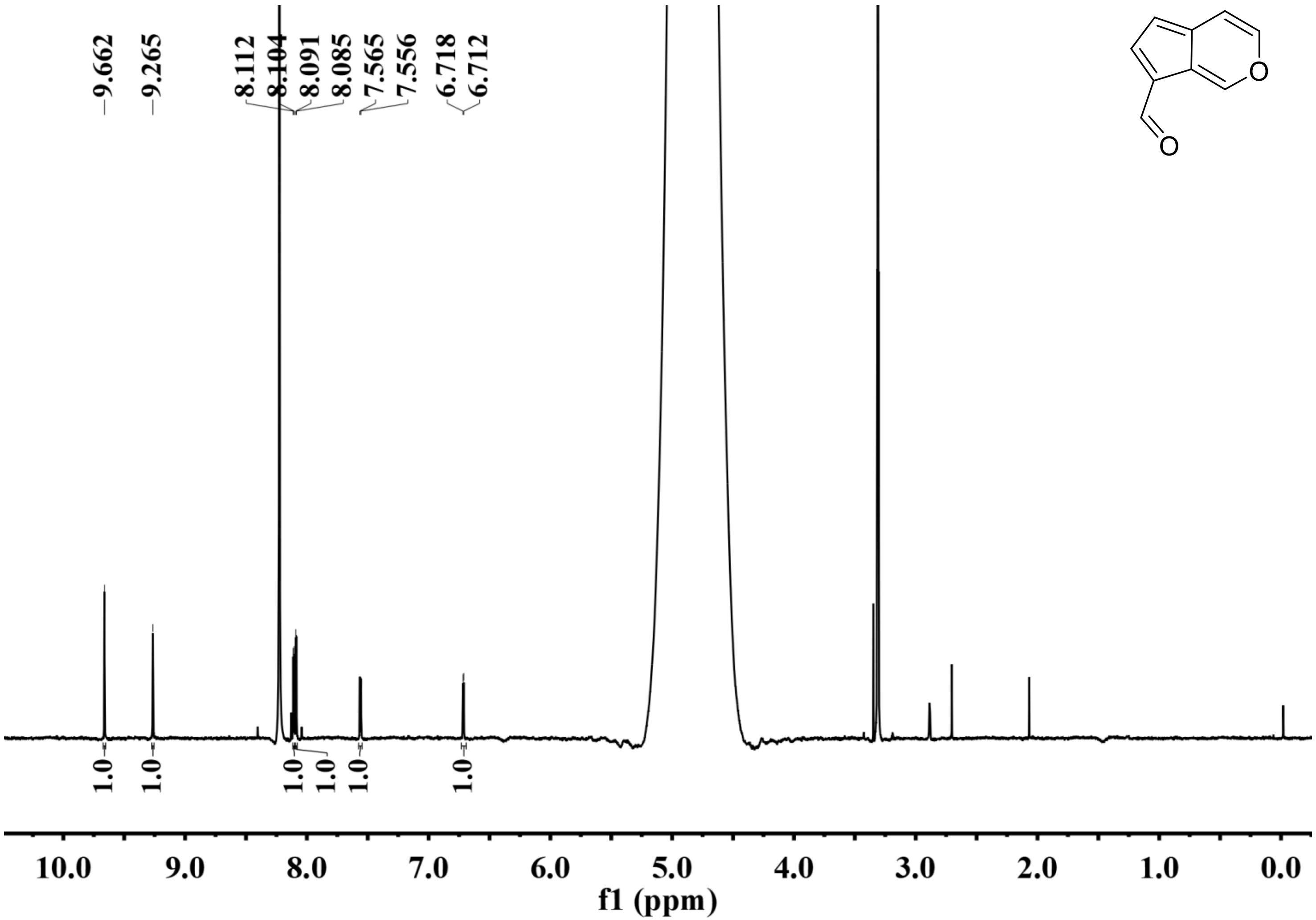

Figure S13. ^1H NMR spectrum of D**3** in CD_3OD (600 MHz)

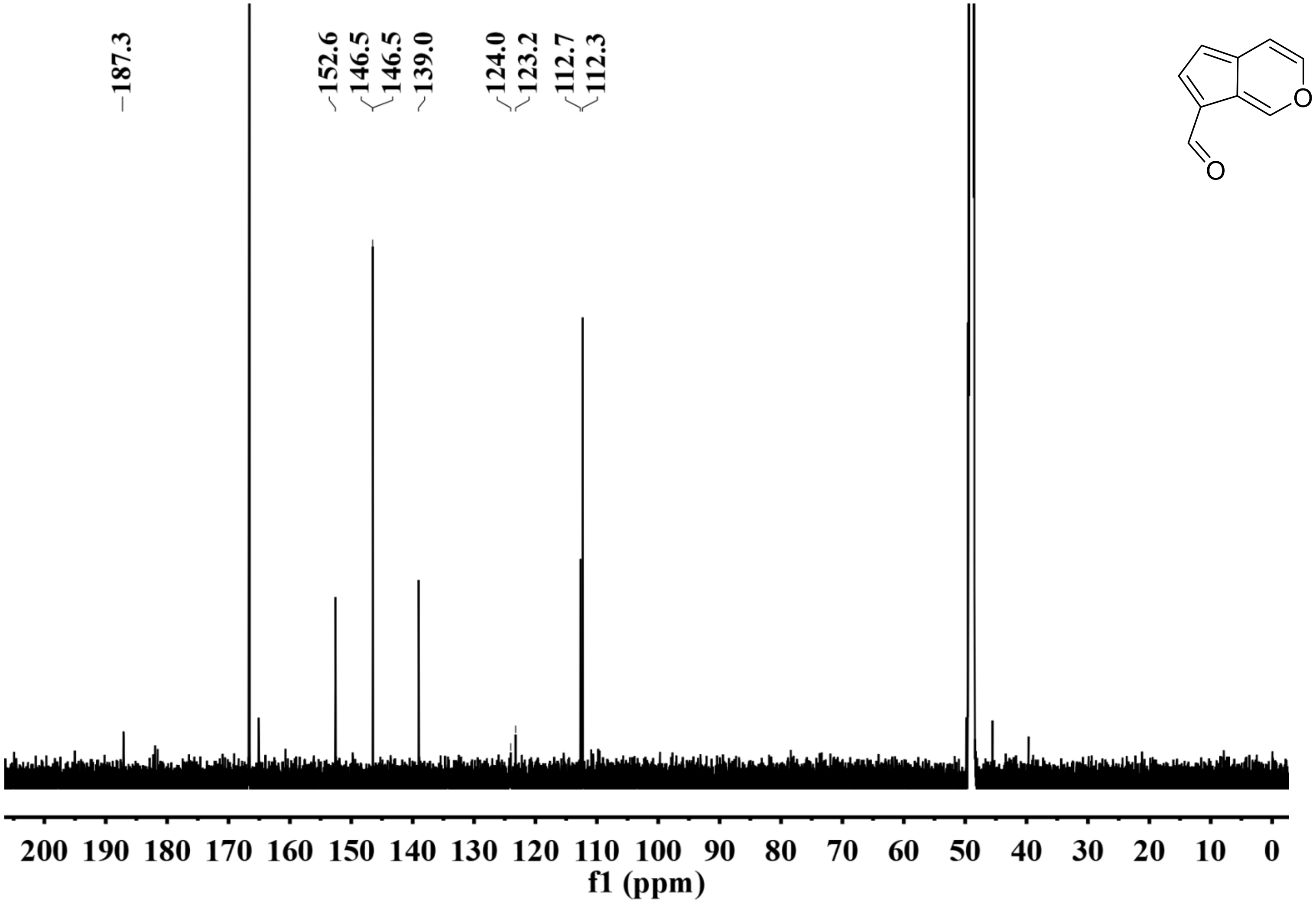

Figure S14. ^{13}C NMR spectrum of D**3** in CD_3OD (150 MHz)

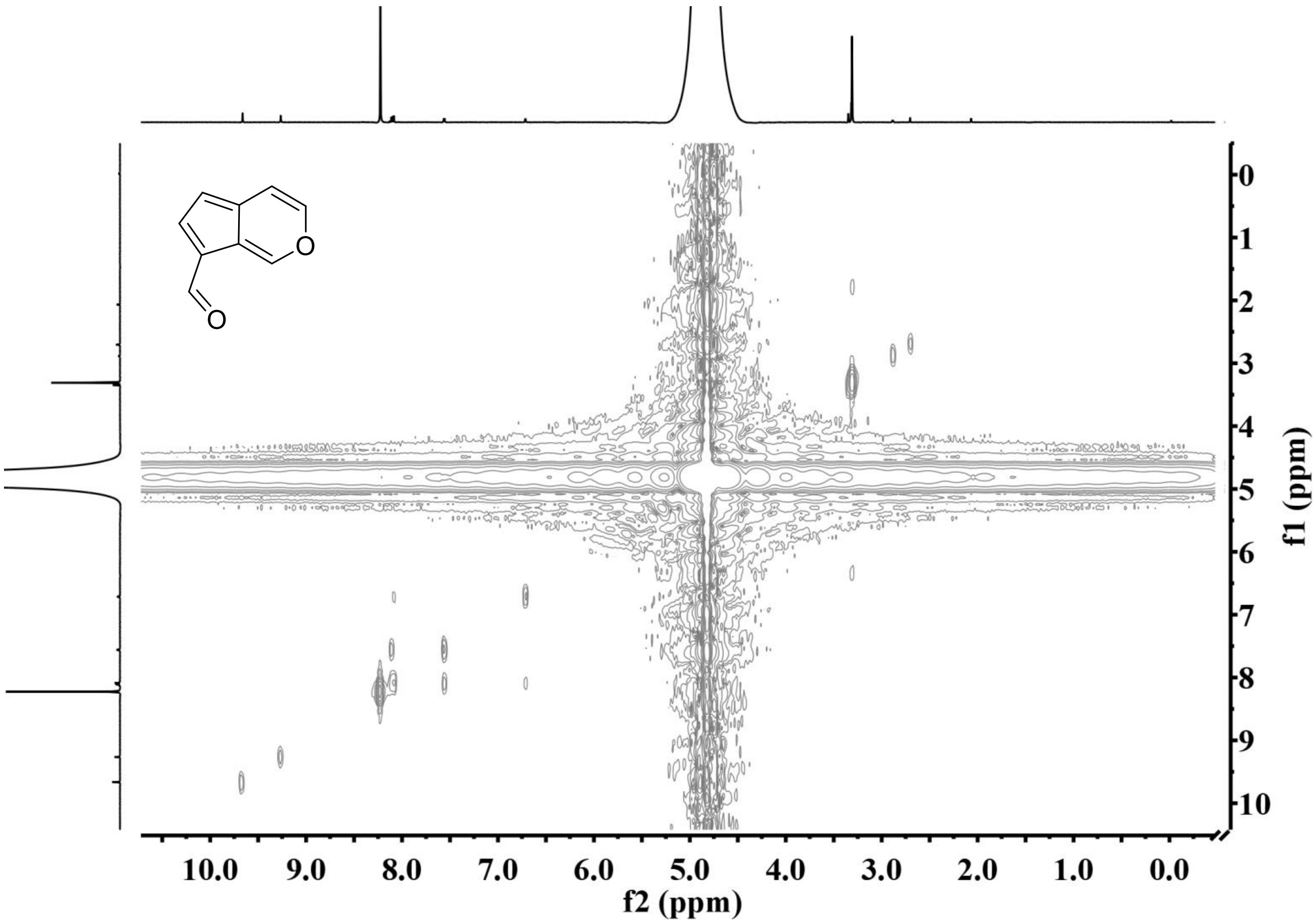

Figure S15. ^{1}H-^{1}H COSY spectrum of D**3** in CD_3OD

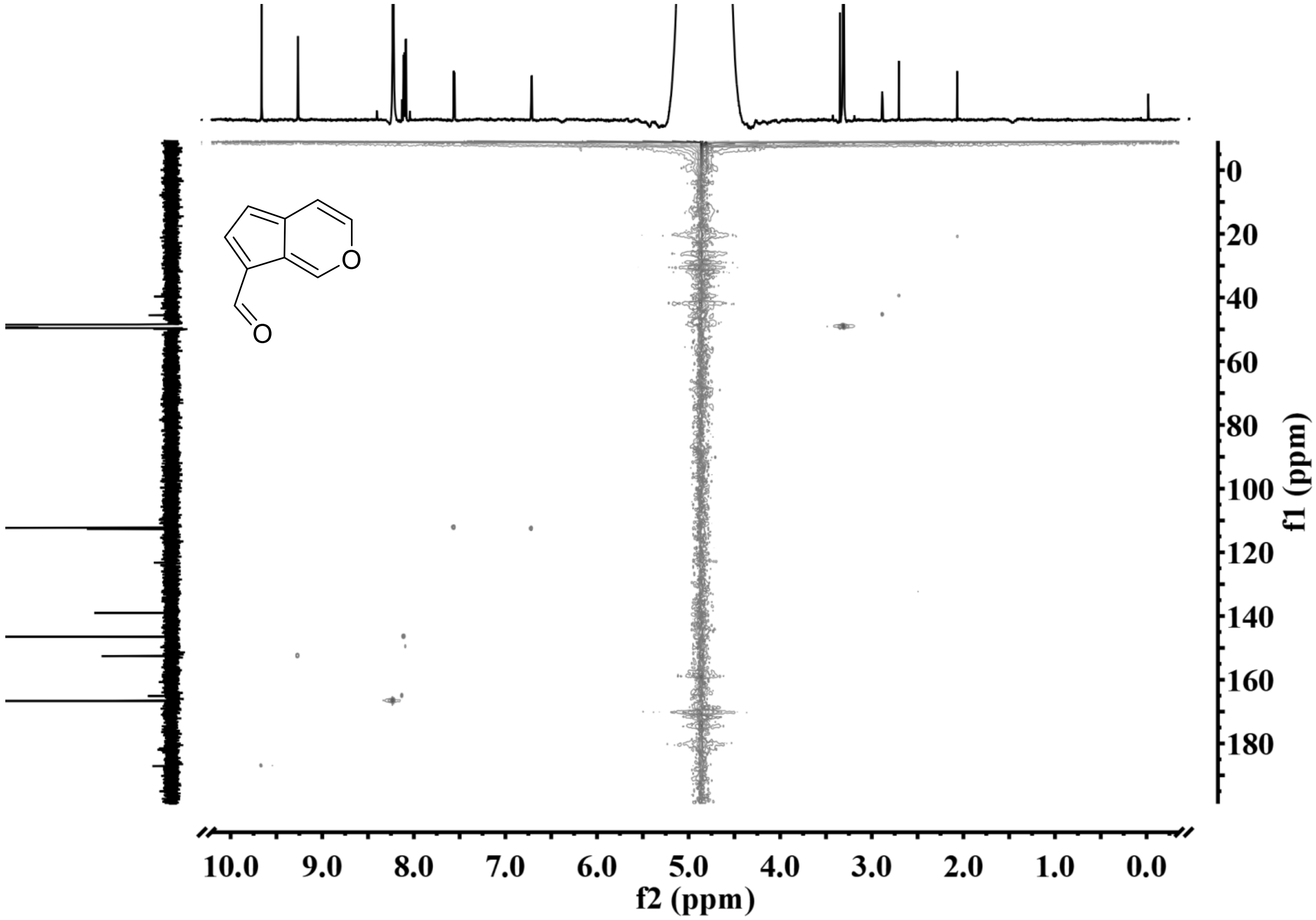

Figure S16. HSQC spectrum of D**3** in CD_3OD

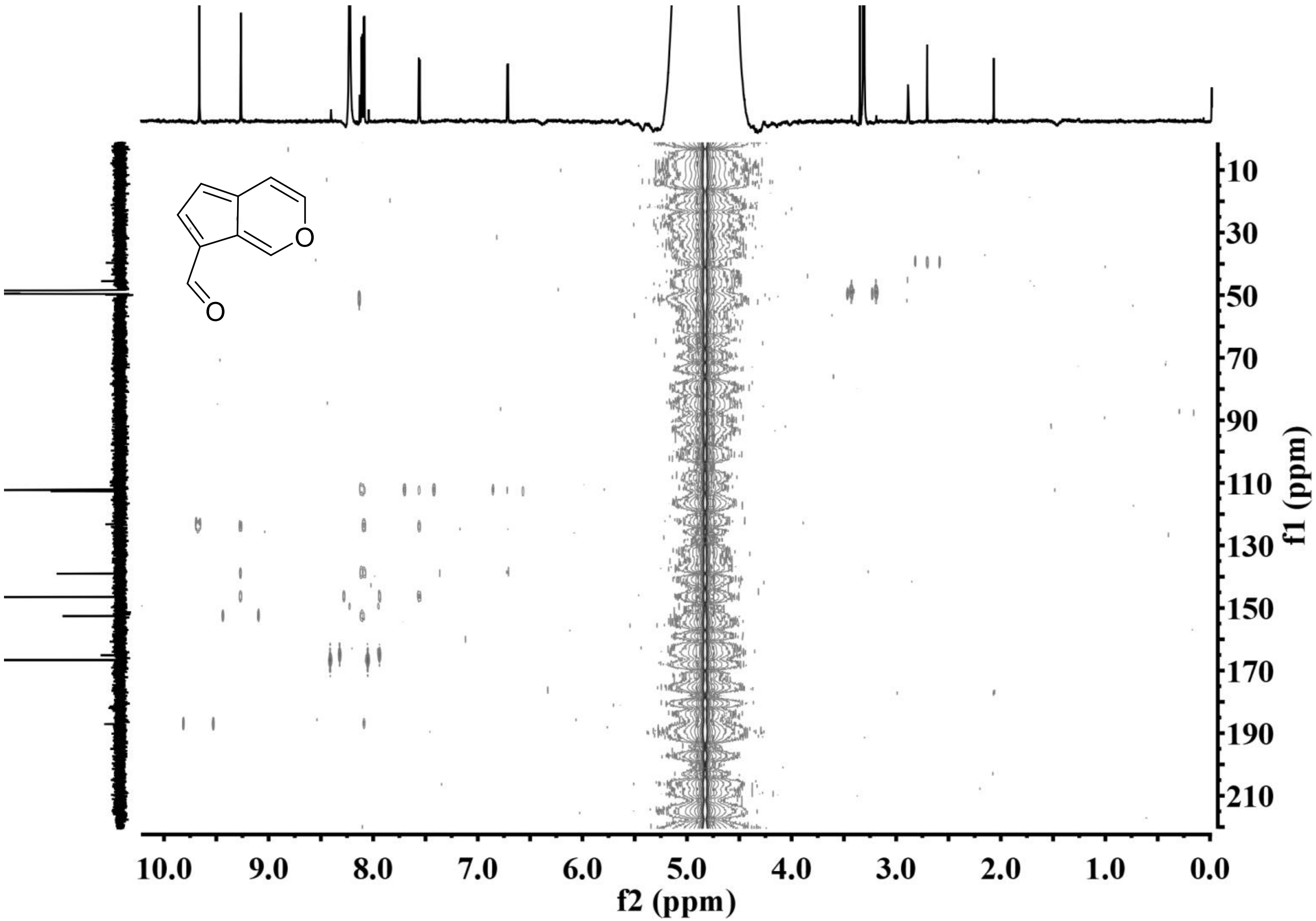

Figure S17. HMBC spectrum of D**3** in CD_3OD

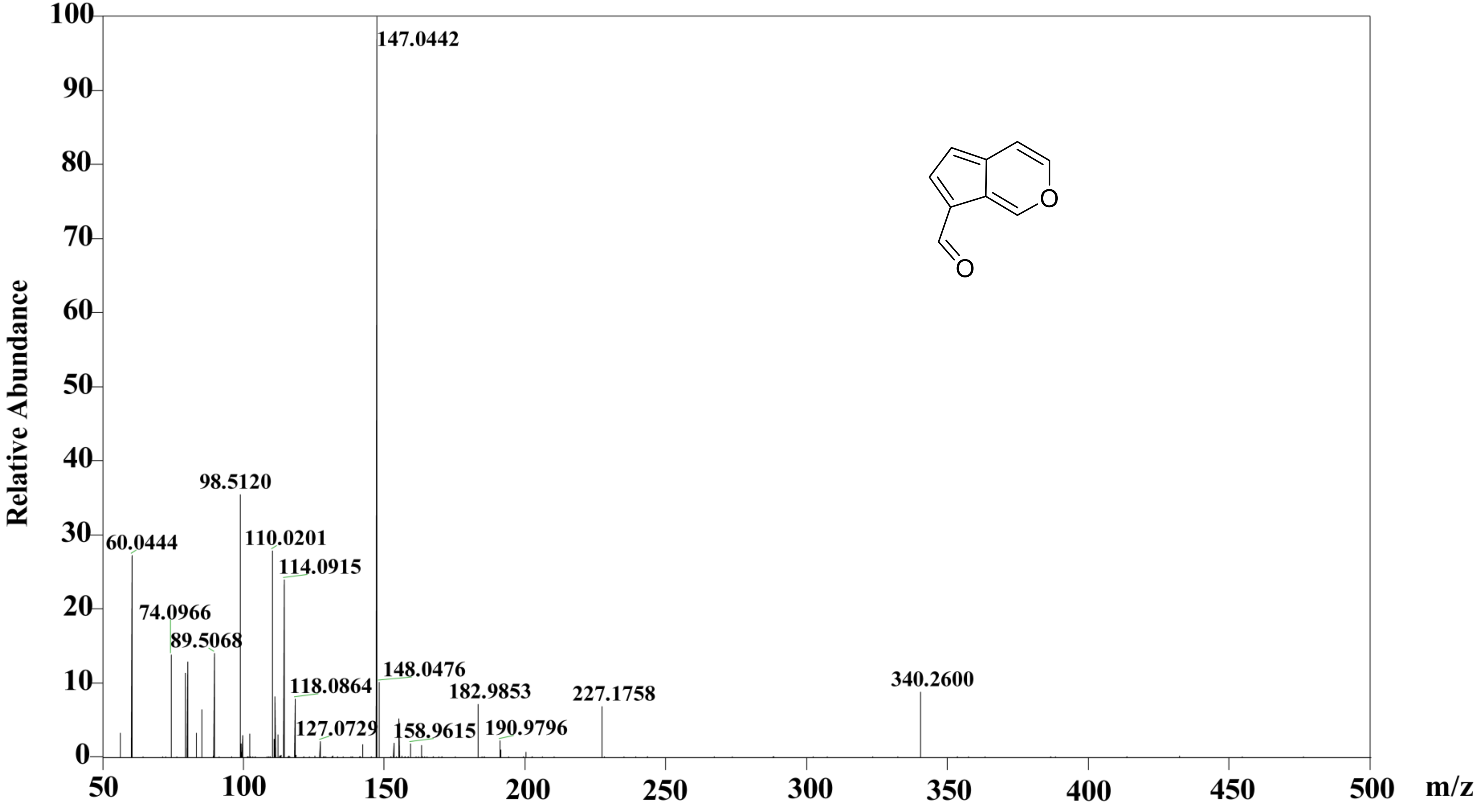

Figure S18. HR-ESI-MS spectrum of D3

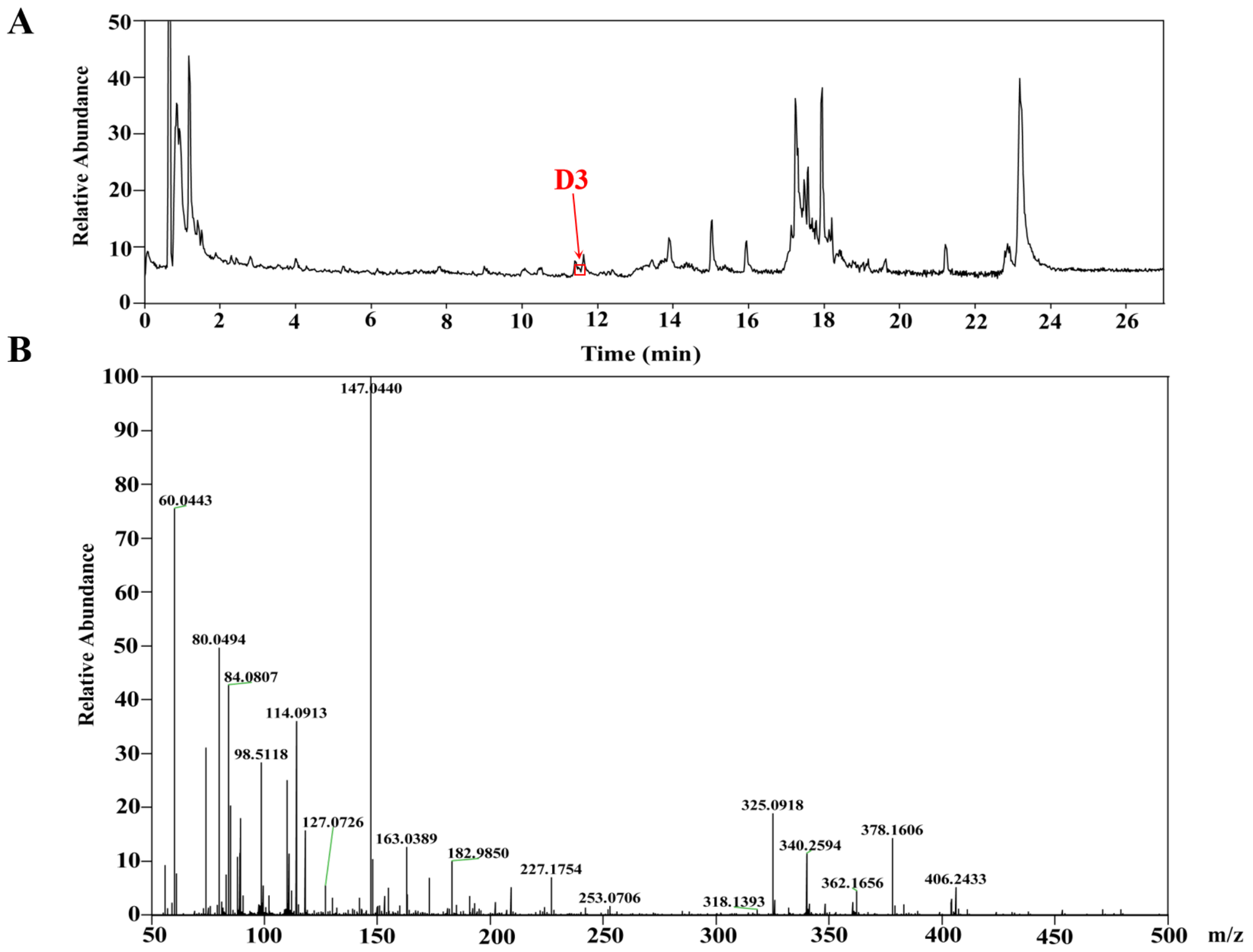

Figure S19. Representative total ion current (TIC) chromatogram of PRR sample in the positive ion mode (A) and MS spectrum of D3 in PRR sample (B)
